# Analysis of adverse drug reactions associated with ravulizumab: a retrospective pharmacovigilance study utilizing the FAERS database

**DOI:** 10.3389/fimmu.2026.1736692

**Published:** 2026-02-25

**Authors:** Yue Zhou, Yutong Wu, Yingchao Su, Zhaoyou Meng

**Affiliations:** Department of Neurology, Second Affiliated Hospital of Army Medical University, Chongqing, China

**Keywords:** FAERS, MG, pharmacovigilance, PNH, ravulizumab

## Abstract

**Background:**

Ravulizumab is a long-acting C5 complement inhibitor that provides sustained suppression of the complement pathway. It is currently approved by the US Food and Drug Administration (FDA) for the treatment of generalized myasthenia gravis, paroxysmal nocturnal hemoglobinuria, and atypical hemolytic uremic syndrome. With its increasing clinical use, concerns regarding Ravulizumab-associated adverse drug reactions have grown.

**Methods:**

This study utilized a dataset extracted from the Adverse Event Reporting System (FAERS) database, comprising adverse event reports from the fourth quarter of 2018 to the second quarter of 2025. Four distinct disproportionality analysis methods—the Reporting Odds Ratio (ROR), Proportional Reporting Ratio (PRR), Multi-item Gamma Poisson Shrinker (MGPS), and Bayesian Confidence Propagation Neural Network (BCPNN)—were applied. Additionally, the time-to-onset profile of adverse events was assessed using the Weibull distribution model.

**Results:**

A total of 9,090 adverse event reports associated with ravulizumab were included in this analysis. Commonly reported adverse events included fatigue, asthenia, headache, malaise, dyspnea, back pain, feeling abnormal, and diplopia. Furthermore, potential adverse events not listed on the drug label were identified, such as anemia, dysphagia, urinary tract infection, and somnolence.

**Conclusion:**

This investigation identified several adverse events associated with ravulizumab and revealed potential adverse reaction signals that were not previously recognized. Healthcare providers may consider these safety signals to more comprehensively assess potential risks in patients during clinical practice.

## 1 Introduction

Ravulizumab is a long-acting C5 complement inhibitor that binds with high affinity and specificity to the human terminal complement protein C5, thereby preventing its cleavage into C5a and C5b and inhibiting the formation of the membrane attack complex (MAC). Dysregulated activation of the complement system is a key pathological mechanism shared by various autoimmune diseases. For instance, in generalized myasthenia gravis (gMG), anti-acetylcholine receptor (AChR) antibodies activate the classical complement pathway, leading to MAC deposition that disrupts the postsynaptic membrane structure at the neuromuscular junction (NMJ) and results in muscle weakness (1–3). Similar complement-mediated tissue damage mechanisms are observed in paroxysmal nocturnal hemoglobinuria (PNH), systemic lupus erythematosus, atypical hemolytic uremic syndrome (aHUS), and acute organ transplant rejection (4–8). Based on this mechanism of action, ravulizumab is currently approved for the treatment of generalized myasthenia gravis, paroxysmal nocturnal hemoglobinuria, atypical hemolytic uremic syndrome, and neuromyelitis optica spectrum disorder (NMOSD).

Structurally, ravulizumab is a humanized monoclonal antibody. Compared to eculizumab, ravulizumab incorporates four key amino acid substitutions in the Fc region. This structural modification significantly enhances its affinity for the neonatal Fc receptor (FcRn) and slows the dissociation rate of the ravulizumab-C5 complex within the endosomal system. Consequently, the plasma half-life of ravulizumab is extended to approximately four times that of eculizumab. This optimization provides the structural basis for the sustained efficacy of ravulizumab and offers convenience for long-term disease management (9).

Ravulizumab initially received approval from FDA in December 2018 for the treatment of PNH. PNH is an ultra-rare, acquired hematopoietic stem cell disorder characterized by a mutation in the PIG-A gene. This mutation results in the production of blood cells that are susceptible to complement-mediated intravascular hemolysis. The clinical manifestations of PNH arise from uncontrolled complement activation, and the complement protein C5 is considered an ideal therapeutic target for inhibiting the complement system in PNH (6).

Ravulizumab received FDA approval in April 2022 for the treatment of gMG in adult patients who are AChR antibody-positive. Myasthenia gravis is an autoimmune disorder characterized by muscle weakness and fatigability, caused by autoantibodies targeting components of the neuromuscular junction (10). In the Phase III CHAMPION-MG trial, intravenous ravulizumab demonstrated statistically significant and clinically meaningful improvements compared to placebo at Week 26, as measured by changes in the Myasthenia Gravis-Activities of Daily Living (MG-ADL) score and the Quantitative Myasthenia Gravis (QMG) score in these patients (11).

Furthermore, ravulizumab received FDA approval in September 2021 for the treatment of aHUS. aHUS is a rare thrombotic microangiopathy (TMA) caused by dysregulation of the complement system, characterized primarily by microangiopathic hemolytic anemia, thrombocytopenia, and acute kidney injury (12–14). Results from a randomized controlled trial investigating ravulizumab for aHUS demonstrated that improvements in hematologic parameters, renal function, and quality of life were sustained for over two years following treatment initiation (15).

As the clinical use of ravulizumab expands, understanding its adverse reaction profile in real-world practice becomes increasingly important. However, the adverse events (AEs) identified in published clinical trials are derived from specific disease populations and thus may only reflect the safety profile within those subsets. Therefore, this study was conducted to address this gap by providing a comprehensive analysis of the safety profile of ravulizumab, thereby offering additional evidence to guide healthcare professionals.

## 2 Materials and methods

**Figure 1:**
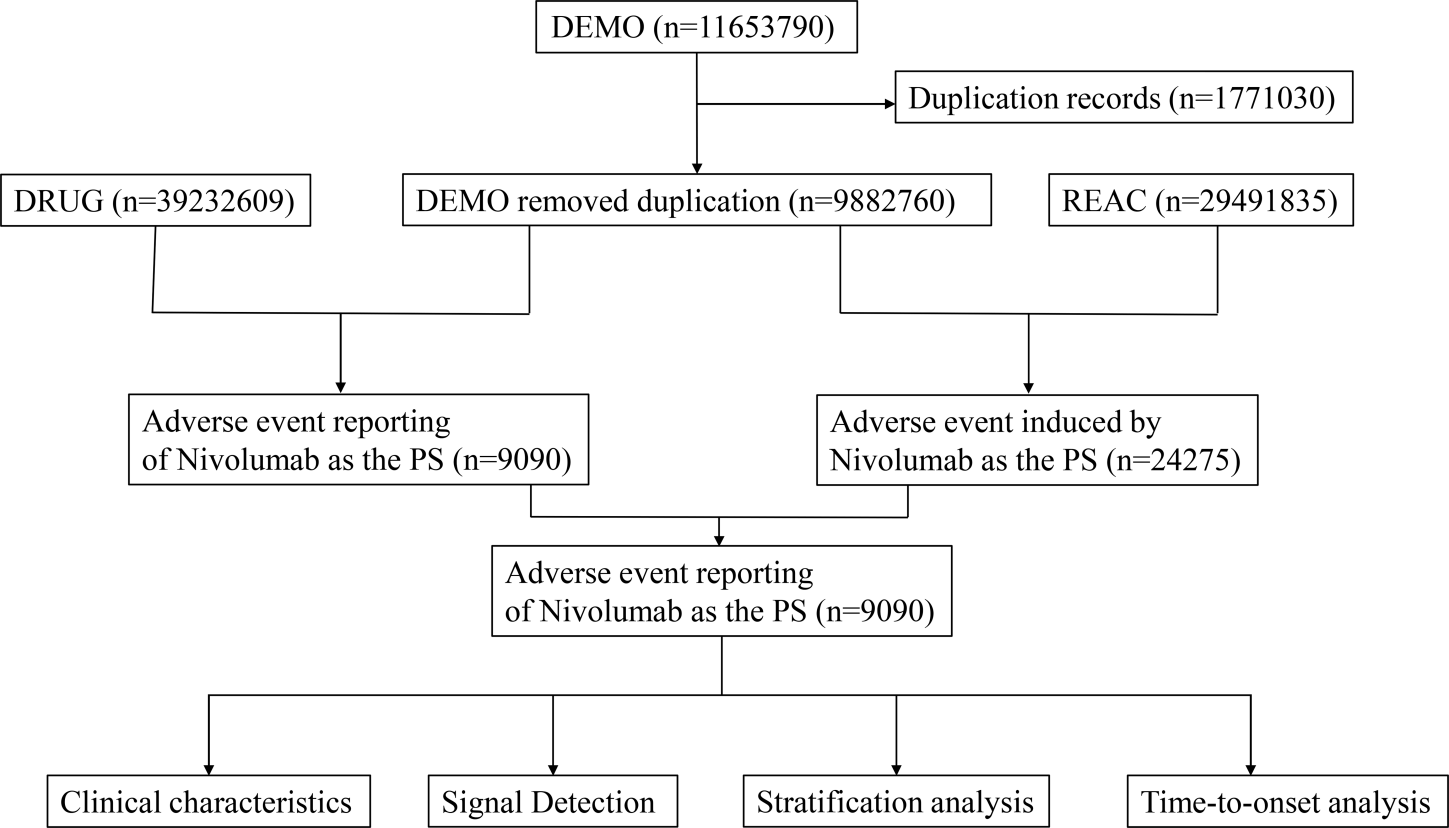
Flow chart.

This study compiled adverse event reports extracted from the US FDA Adverse Event Reporting System (FAERS) database from the fourth quarter of 2018 to the second quarter of 2025. Reports listing ravulizumab as the primary suspect (PS) drug were included. Data extraction and cleaning were performed using R software version 4.4.1. The initial dataset contained 11,653,790 reports. Following FDA recommendations (16), 1,771,030 duplicate reports were excluded. The PRIMARYID, CASEID, and FDA_DT fields were extracted from the DEMO table of the raw data and subsequently sorted. For reports sharing the same CASEID, the entry with the latest FDA_DT was retained. For reports with identical CASEID and FDA_DT values, the entry with the highest PRIMARYID was kept. Adverse events were coded using the Medical Dictionary for Regulatory Activities (MedDRA), version 27.0 (17), based on Preferred Terms (PT) and System Organ Classes (SOC). **Figure 1** illustrates the flowchart for identifying ravulizumab-associated adverse events in the FAERS database.

### 2.1 Data analysis

Multiple disproportionality analysis methods were employed to evaluate significant associations between ravulizumab and AEs, including the reporting odds ratio (ROR) (18–20), proportional reporting ratio (PRR) (21), multi-item gamma Poisson shrinker (MGPS) (22), and Bayesian confidence propagation neural network (BCPNN) (23). An AE was considered a potential safety signal if it exceeded the positive threshold in at least one of these methods. The 2×2 contingency matrix is detailed in **Supplementary Table S1**, and the specific algorithms used for signal detection are further described in **Supplementary Table S2**. Additionally, the time-to-onset of AEs was modeled using a Weibull distribution.

## 3 Results

### 3.1 Baseline characteristics of AEs and the reported population

A total of 9,090 adverse event reports associated with ravulizumab were included in this analysis. **Table 1** summarizes the baseline characteristics of these reports. Females (2,983, 32.8%) accounted for a slightly higher proportion of reports than males (2,655, 29.2%). Among the reported cases, adults aged 18–65 years (1,014, 11.2%) constituted the largest age group, followed by individuals over 65 years (820, 9.0%). As anticipated, the primary indication for ravulizumab use was gMG (3,351, 36.9%), with PNH(1,951, 21.5%) and aHUS(965, 10.6%) being the other leading indications. According to the FAERS data, the most frequently reported serious outcome was hospitalization (1,271, 14.0%), followed by death, life-threatening events, and disability. The United States (7,744, 85.2%) submitted the highest number of reports, followed by Japan (478, 5.3%). Consumers were the primary reporters of AEs(4,748, 52.2%). Following the FDA approval of ravulizumab for gMG in 2022, the number of associated AE reports increased rapidly: 395 cases were recorded in 2022, which rose to 1,403 cases (15.4% of the total) in 2023, and further climbed to 4,476 cases (49.2% of the total) in 2024. This trend reflects the expanding real-world utilization of ravulizumab.

**Table 1:** Clinical characteristics of ravulizumab adverse event reports from the FAERS database (Q4 2018 – Q2 2025).

Characteristics	Case numbers	Case proportion (%)
Number of events	9090	
Gender		
Male	2655	29.2%
Female	2983	32.8%
Unknown	3452	38.0%
Age		
<18	172	1.9%
18-65	1014	11.2%
65-85	820	9.0%
Unknown	7084	77.9%
Top 3 indication		
Myasthenia gravis	3351	36.9%
Paroxysmal nocturnal hemoglobinuria	1951	21.5%
atypical hemolytic uremic syndrome	965	10.6%
Outcome		
Hospitalization	1271	14.0%
Death	454	5.0%
Life-Threatening	60	0.7%
Disability	14	0.2%
Top 5 reported countries		
United States	7744	85.2%
Japan	478	5.3%
United Kingdom	151	1.7%
Germany	125	1.4%
Canada	87	1.0%
Reporter		
Consumer	4748	52.2%
Physician	1884	20.7%
Health Professional	432	4.8%
Pharmacist	282	3.1%
Reporting year		
2019	525	5.8%
2020	401	4.4%
2021	388	4.3%
2022	395	4.3%
2023	1403	15.4%
2024	4476	49.2%
2025	1502	16.5%

### 3.2 Signal detection at the SOC level

**Figure 2:**
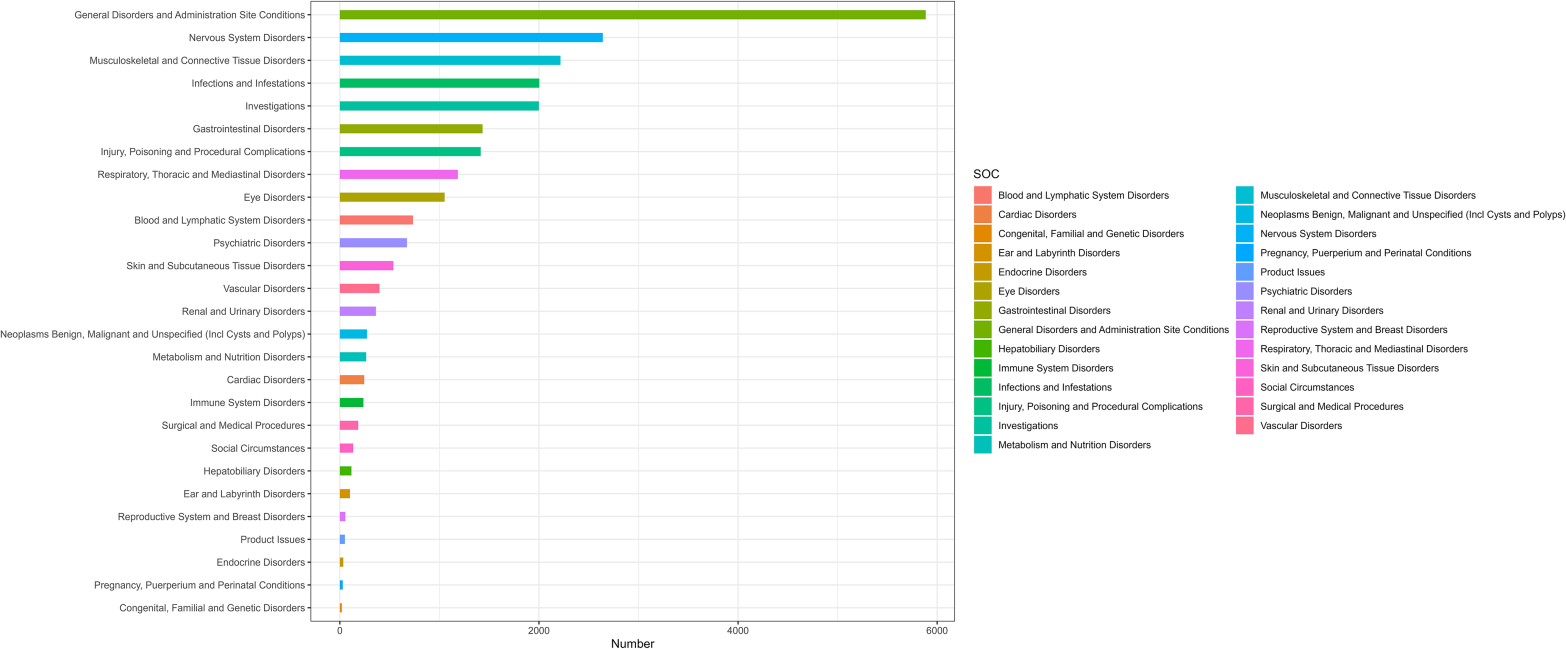
SOC distribution diagram.

**Table 2** presents the AEs associated with ravulizumab across all 27 SOCs, while **Figure 2** depict the signal strengths for ravulizumab at the SOC level within the FAERS database. The most frequently reported SOC was general disorders and administration site conditions [n = 5,885; ROR (95% CI) = 1.5 (1.46-1.55)], whereas the strongest signal strength was observed for eye disorders [n = 1,053; ROR (95% CI) = 2.28 (2.14-2.42)]. Several additional SOCs demonstrated robust signals, including musculoskeletal and connective tissue disorders [n = 2,216; ROR (95% CI) = 1.9 (1.82-1.99)], blood and lymphatic system disorders [n = 734; ROR (95% CI) = 1.8 (1.68-1.94)], and nervous system disorders [n = 2,641; ROR (95% CI) = 1.54 (1.48-1.60)]. lack of a statistically significant signal, defined by the lower limit of the ROR 95% CI being below 1, was observed for SOCs such as product issues, reproductive system and breast disorders, hepatobiliary disorders, cardiac disorders, and skin and subcutaneous tissue disorders.

**Table 2:** Signal strength of ravulizumab AEs across System Organ Classes (SOC) in the FAERS database.

System Organ Class (SOC)	Case numbers	ROR (95%CI)	PRR(χ^2^)	EBGM(EBGM05)	IC(IC025)
general disorders and administration site conditions*	5885	1.5 (1.46 - 1.55)	1.38 (753.84)	1.38 (1.34)	0.47 (0.42)
nervous system disorders*	2641	1.54 (1.48 - 1.6)	1.48 (444.79)	1.48 (1.42)	0.57 (0.51)
musculoskeletal and connective tissue disorders*	2216	1.9 (1.82 - 1.99)	1.82 (861.79)	1.82 (1.74)	0.86 (0.8)
infections and infestations*	2003	1.51 (1.45 - 1.59)	1.47 (321.32)	1.47 (1.41)	0.56 (0.49)
investigations*	1999	1.47 (1.41 - 1.54)	1.43 (276.25)	1.43 (1.37)	0.52 (0.45)
gastrointestinal disorders	1434	0.72 (0.69 - 0.76)	0.74 (142.56)	0.74 (0.7)	-0.43 (-0.51)
injury, poisoning and procedural complications	1415	0.44 (0.41 - 0.46)	0.47 (970)	0.47 (0.44)	-1.09 (-1.17)
respiratory, thoracic and mediastinal disorders*	1185	1.08 (1.02 - 1.14)	1.07 (6.09)	1.07 (1.01)	0.1 (0.01)
eye disorders*	1053	2.28 (2.14 - 2.42)	2.22 (719.82)	2.22 (2.09)	1.15 (1.06)
blood and lymphatic system disorders*	734	1.8 (1.68 - 1.94)	1.78 (254.55)	1.78 (1.65)	0.83 (0.72)
psychiatric disorders	674	0.52 (0.48 - 0.56)	0.53 (290.52)	0.53 (0.49)	-0.91 (-1.02)
skin and subcutaneous tissue disorders	538	0.36 (0.34 - 0.4)	0.38 (581.23)	0.38 (0.35)	-1.4 (-1.52)
vascular disorders	398	0.88 (0.79 - 0.97)	0.88 (6.73)	0.88 (0.8)	-0.19 (-0.33)
renal and urinary disorders	362	0.76 (0.69 - 0.84)	0.76 (27.02)	0.76 (0.69)	-0.39 (-0.54)
neoplasms benign, malignant and unspecified (incl cysts and polyps)	272	0.32 (0.29 - 0.37)	0.33 (377.5)	0.33 (0.3)	-1.59 (-1.76)
metabolism and nutrition disorders	265	0.55 (0.49 - 0.62)	0.56 (94.99)	0.56 (0.49)	-0.84 (-1.02)
cardiac disorders	244	0.5 (0.44 - 0.57)	0.51 (119.77)	0.51 (0.45)	-0.98 (-1.16)
immune system disorders	236	0.79 (0.69 - 0.9)	0.79 (13.16)	0.79 (0.7)	-0.34 (-0.52)
surgical and medical procedures	184	0.5 (0.44 - 0.58)	0.51 (89.57)	0.51 (0.44)	-0.98 (-1.19)
social circumstances	133	1.13 (0.95 - 1.34)	1.13 (2.04)	1.13 (0.95)	0.18 (-0.07)
hepatobiliary disorders	116	0.55 (0.46 - 0.66)	0.56 (41.64)	0.56 (0.46)	-0.85 (-1.11)
ear and labyrinth disorders	101	1.01 (0.83 - 1.23)	1.01 (0.02)	1.01 (0.83)	0.02 (-0.27)
reproductive system and breast disorders	54	0.34 (0.26 - 0.44)	0.34 (69.82)	0.34 (0.26)	-1.56 (-1.93)
product issues	50	0.1 (0.08 - 0.14)	0.11 (381.21)	0.11 (0.08)	-3.23 (-3.61)
endocrine disorders	34	0.51 (0.37 - 0.72)	0.51 (15.76)	0.51 (0.37)	-0.96 (-1.43)
pregnancy, puerperium and perinatal conditions	29	0.33 (0.23 - 0.48)	0.33 (38.83)	0.33 (0.23)	-1.58 (-2.08)
congenital, familial and genetic disorders	20	0.33 (0.21 - 0.51)	0.33 (27.52)	0.33 (0.21)	-1.61 (-2.19)

Asterisks (*) indicate statistically significant signals in algorithm; ROR, reporting odds ratio; PRR, proportional reporting ratio; EBGM, empirical Bayesian geometric mean; EBGM05, the lower limit of the 95% CI of EBGM; IC, information component; IC025, the lower limit of the 95% CI of the IC; CI, confidence interval; AEs, adverse events.

### 3.3 Signal detection at the PT level

We systematically categorized all adverse events associated with ravulizumab based on frequency, with the results detailed in **Table 3**. The top 10 most frequently reported PTs were fatigue [n = 1,317; ROR (95%CI) = 4.49 (4.24-4.74)], asthenia [n = 668; ROR (95%CI) = 5.14 (4.76-5.56)], headache [n = 589; ROR (95%CI) = 2.69 (2.48-2.92)], dyspnea [n = 346; ROR (95%CI) = 1.69 (1.52-1.88)], malaise [n = 310; ROR (95%CI) = 2.03 (1.82-2.27)], back pain [n = 297; ROR (95%CI) = 3.65 (3.25-4.09)], feeling abnormal [n = 272; ROR (95%CI) = 3.38 (3-3.81)], diplopia [n = 267; ROR (95%CI) = 31.24 (27.65-35.3)], and nausea [n = 242; ROR (95%CI) = 0.87 (0.77-0.99)], anemia [n = 124; ROR (95%CI) = 1.9 (1.59-2.26)]. Several of these PTs, including fatigue, asthenia, headache, malaise, dyspnea, back pain, feeling abnormal, and diplopia, are listed in the official drug label. Potential adverse reactions not currently mentioned in the prescribing information were also identified, such as anemia, dysphagia, urinary tract infection, and somnolence.

**Table 3:** Top 50 frequency of adverse events at the PT level for ravulizumab.

PT	Case numbers	ROR(95%CI)	PRR(χ^2^)	EBGM(EBGM05)	IC(IC025)
fatigue*	1317	4.49 (4.24 - 4.74)	4.3 (3362.48)	4.29 (4.05)	2.1 (2.01)
asthenia*	668	5.14 (4.76 - 5.56)	5.03 (2159.89)	5.01 (4.64)	2.33 (2.2)
headache*	589	2.69 (2.48 - 2.92)	2.65 (608.37)	2.64 (2.44)	1.4 (1.28)
drug ineffective	488	0.88 (0.8 - 0.96)	0.88 (8)	0.88 (0.81)	-0.18 (-0.31)
off label use	424	0.95 (0.86 - 1.04)	0.95 (1.29)	0.95 (0.86)	-0.08 (-0.22)
muscular weakness*	381	10.2 (9.22 - 11.29)	10.06 (3087.54)	9.98 (9.02)	3.32 (3.14)
dyspnea*	346	1.69 (1.52 - 1.88)	1.68 (95.48)	1.68 (1.51)	0.75 (0.59)
malaise*	310	2.03 (1.82 - 2.27)	2.02 (159.97)	2.02 (1.8)	1.01 (0.84)
back pain*	297	3.65 (3.25 - 4.09)	3.61 (562.01)	3.61 (3.22)	1.85 (1.67)
hemoglobin decreased*	291	8.38 (7.46 - 9.41)	8.29 (1856.32)	8.24 (7.34)	3.04 (2.84)
therapeutic response shortened*	288	14.96 (13.31 - 16.81)	14.79 (3661.55)	14.62 (13.01)	3.87 (3.63)
myasthenia gravis*	281	75.26 (66.67 - 84.95)	74.4 (19175.33)	70.16 (62.16)	6.13 (5.64)
pain	278	1.06 (0.94 - 1.19)	1.05 (0.79)	1.05 (0.94)	0.08 (-0.1)
feeling abnormal*	272	3.38 (3 - 3.81)	3.35 (449.19)	3.35 (2.97)	1.74 (1.55)
diplopia*	267	31.24 (27.65 - 35.3)	30.91 (7538.99)	30.17 (26.7)	4.92 (4.59)
death	249	0.73 (0.65 - 0.83)	0.74 (23.98)	0.74 (0.65)	-0.44 (-0.62)
nausea	242	0.87 (0.77 - 0.99)	0.88 (4.33)	0.88 (0.77)	-0.19 (-0.38)
diarrhea	237	0.93 (0.82 - 1.06)	0.93 (1.24)	0.93 (0.82)	-0.1 (-0.29)
arthralgia*	213	1.28 (1.11 - 1.46)	1.27 (12.56)	1.27 (1.11)	0.35 (0.15)
dizziness*	203	1.2 (1.04 - 1.38)	1.2 (6.63)	1.2 (1.04)	0.26 (0.05)
eyelid ptosis*	201	59.21 (51.36 - 68.25)	58.72 (10880.15)	56.06 (48.63)	5.81 (5.25)
symptom recurrence*	200	52.37 (45.43 - 60.36)	51.94 (9583.99)	49.85 (43.25)	5.64 (5.12)
covid-19*	199	1.45 (1.26 - 1.66)	1.44 (27.2)	1.44 (1.25)	0.53 (0.32)
hemolysis*	199	85.6 (74.1 - 98.89)	84.91 (15424.14)	79.42 (68.75)	6.31 (5.62)
gait disturbance*	197	2.89 (2.51 - 3.33)	2.88 (241.07)	2.87 (2.49)	1.52 (1.3)
pain in extremity*	192	1.9 (1.65 - 2.19)	1.89 (81.34)	1.89 (1.64)	0.92 (0.71)
dysphagia*	189	5.82 (5.04 - 6.71)	5.78 (744.2)	5.76 (4.99)	2.52 (2.28)
nasopharyngitis*	170	2.26 (1.94 - 2.63)	2.25 (118.28)	2.25 (1.93)	1.17 (0.94)
fall*	161	1.35 (1.15 - 1.57)	1.34 (14.19)	1.34 (1.15)	0.43 (0.19)
pyrexia*	160	1.27 (1.09 - 1.48)	1.27 (9.11)	1.27 (1.09)	0.34 (0.11)
illness*	153	2.25 (1.92 - 2.64)	2.24 (105.4)	2.24 (1.91)	1.16 (0.92)
pneumonia	144	1.13 (0.96 - 1.34)	1.13 (2.27)	1.13 (0.96)	0.18 (-0.06)
urinary tract infection*	133	1.99 (1.67 - 2.35)	1.98 (64.59)	1.98 (1.67)	0.98 (0.72)
weight decreased*	130	1.23 (1.04 - 1.46)	1.23 (5.61)	1.23 (1.03)	0.3 (0.04)
cough	129	1.12 (0.94 - 1.33)	1.12 (1.7)	1.12 (0.94)	0.16 (-0.09)
somnolence*	129	1.83 (1.53 - 2.17)	1.82 (47.78)	1.82 (1.53)	0.86 (0.6)
infection*	129	2.14 (1.8 - 2.55)	2.13 (77.89)	2.13 (1.79)	1.09 (0.83)
muscle spasms*	128	2.11 (1.78 - 2.51)	2.11 (74.53)	2.11 (1.77)	1.07 (0.81)
anemia*	124	1.9 (1.59 - 2.26)	1.89 (52.31)	1.89 (1.59)	0.92 (0.65)
myalgia*	124	2.25 (1.88 - 2.68)	2.24 (85.29)	2.24 (1.88)	1.16 (0.89)
blood pressure increased*	121	2.05 (1.71 - 2.45)	2.04 (64.25)	2.04 (1.7)	1.03 (0.75)
condition aggravated	120	0.85 (0.71 - 1.02)	0.85 (3.16)	0.85 (0.71)	-0.23 (-0.49)
general physical health deterioration*	110	2.32 (1.93 - 2.8)	2.32 (82.48)	2.32 (1.92)	1.21 (0.92)
balance disorder*	104	3.52 (2.91 - 4.27)	3.51 (186.74)	3.51 (2.89)	1.81 (1.49)
vomiting	101	0.62 (0.51 - 0.75)	0.62 (23.42)	0.62 (0.51)	-0.69 (-0.97)
blood lactate dehydrogenase increased*	101	23.87 (19.59 - 29.08)	23.77 (2161.52)	23.34 (19.16)	4.54 (3.97)
platelet count decreased*	100	2.38 (1.96 - 2.9)	2.38 (79.94)	2.38 (1.95)	1.25 (0.94)
visual impairment*	100	1.95 (1.6 - 2.38)	1.95 (46.21)	1.95 (1.6)	0.96 (0.66)
speech disorder*	98	5.65 (4.63 - 6.89)	5.63 (371.52)	5.61 (4.6)	2.49 (2.13)
stress*	97	3.38 (2.77 - 4.13)	3.37 (161.56)	3.37 (2.76)	1.75 (1.42)

Asterisks (*) indicate statistically significant signals in algorithm; ROR, reporting odds ratio; PRR, proportional reporting ratio; EBGM, empirical Bayesian geometric mean; EBGM05, the lower limit of the 95% CI of EBGM; IC, information component; IC025, the lower limit of the 95% CI of the IC; CI, confidence interval; PT, preferred term.

### 3.4 Subgroup analysis

**Supplementary Tables 3–9** provide detailed results of the subgroup analyses related to ravulizumab. In the gender-based analysis, fatigue was the most frequently reported adverse event in both males [n = 433; ROR (95%CI) = 5.39 (4.89-5.94)] and females [n = 535; ROR (95%CI) = 5 (4.58-5.46)]. Notably, asthenia was more commonly reported in males [n = 261; ROR (95%CI) = 6.31 (5.58-7.14)], whereas headache was more prevalent in females [n = 287; ROR (95%CI) = 3.17 (2.81-3.56)]. Within age subgroups, individuals under 18 years of age exhibited a higher incidence of hemolysis [n = 13; ROR (95%CI) = 161.49 (91.69-284.44)]. In contrast, fatigue was the most common adverse event reported among adults (18 years and older) [18–65 years: n = 132, ROR (95%CI) = 3.43 (2.88-4.08); over 65 years: n = 112, ROR (95%CI) = 3.56 (2.94-4.3)]. An analysis by reporter type revealed that consumers most frequently reported adverse events such as fatigue [n = 938; ROR (95%CI) = 5.08 (4.75-5.43)] and headache [n = 424; ROR (95%CI) = 3.13 (2.84-3.44)]. Conversely, healthcare professionals primarily reported issues including off-label use [n = 302; ROR (95%CI) = 2035 (2.09-2.63)] and drug ineffective [n = 138; ROR (95%CI) = 1.22 (1.03-1.44)].

### 3.5 Sensitivity analysis

Based on the baseline data from the FAERS database, the primary indications for ravulizumab are PNH, gMG and aHUS. To minimize potential confounding from concomitant medications used to treat these conditions, we excluded reports involving specific drugs, including eculizumab, prednisone, pyridostigm bromide, and intravenous immunoglobulin. Following the exclusion of cases requiring concurrent treatment with these medications, a total of 7,284 case reports encompassing 17,976 adverse events were retained for analysis. The primary adverse events reported in this refined dataset included fatigue, asthenia, headache, dyspnea, and back pain. Detailed results are provided in **Supplementary Table 10**.

### 3.6 Analysis of time-to-onset of adverse events and Weibull distribution modeling for ravulizumab

**Figure 3:**
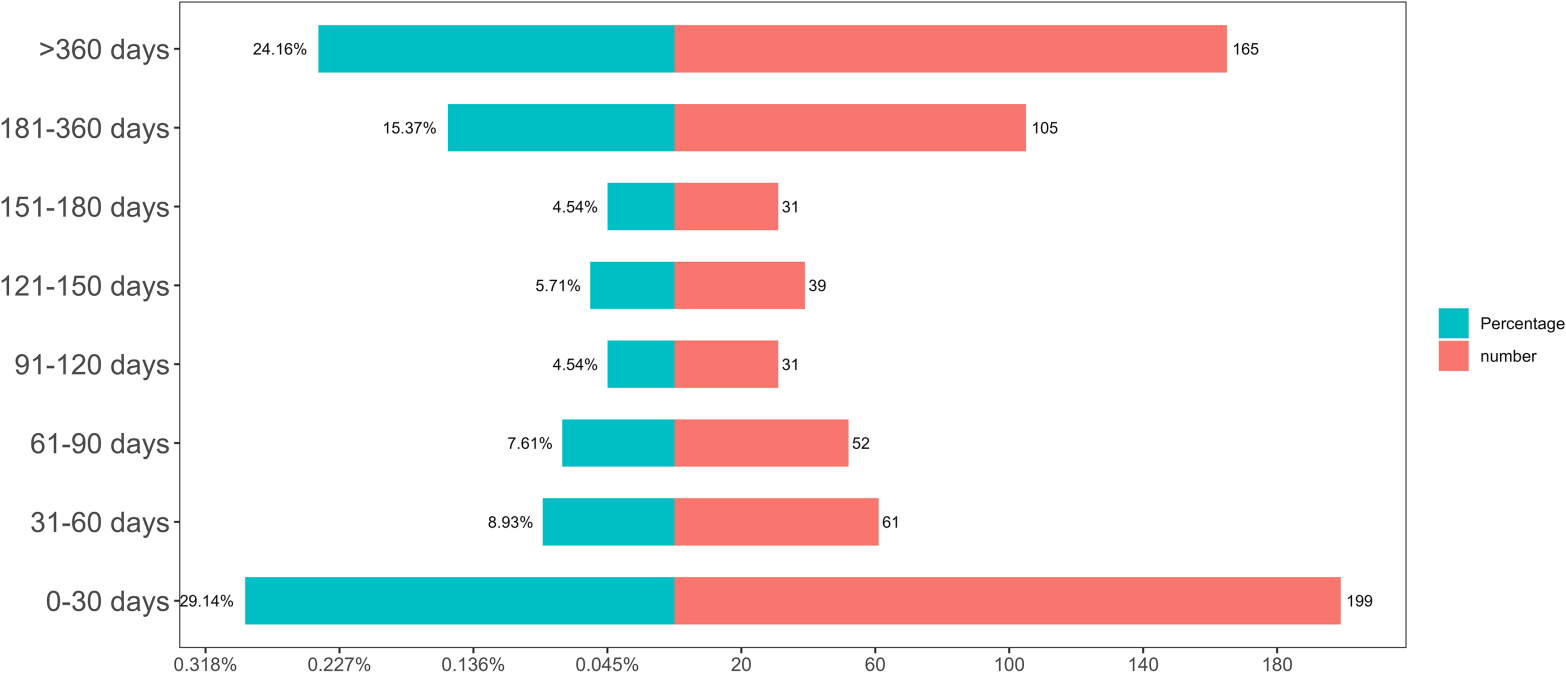
Adverse event induction time.

As shown in **Figure 3**, the majority of Ravulizumab-associated AEs occurred within the first 30 days after treatment initiation. Furthermore, the Weibull distribution analysis revealed an early failure pattern, with the specific distribution parameters and detailed time-to-onset (TTO) data of the eligible cases all presented in **Table 4**.

**Table 4:** Time to onset of ravulizumab-associated adverse events and Weibull distribution analysis.

Drug	TTO(days)		Weibull distribution		
	Case reports	Median(d)(IQR)	Scale parameter: α(95%CI)	Shape parameter: β(95%CI)	Type
Ravulizumab	683	120(19, 349)	198.63(173.62, 223.64)	0.63(0.59, 0.66)	Early failure

TTO, time to onset; CI, confidence interval; IQR, interquartile range.

## 4 Discussion

This study analyzed adverse events associated with ravulizumab recorded in the FAERS database from the fourth quarter of 2018, following its initial FDA approval and subsequent post-marketing phase. The findings not only confirm various adverse reactions previously documented in the prescribing information—including fatigue, asthenia, headache, malaise, dyspnea, back pain, feeling abnormal, and diplopia—but also identify additional AEs not currently included in the label, such as anemia, dysphagia, urinary tract infection, and somnolence.

Our study demonstrates that the top 10 most frequently reported AEs associated with ravulizumab are largely consistent with those listed in its prescribing information, thereby confirming that fatigue, asthenia, and headache are common AEs for this medication.

Ravulizumab shares a mechanism of action similar to eculizumab, specifically binding to the complement protein C5. This binding inhibits the generation of the anaphylatoxin C5a and the terminal complement component C5b, which is essential for forming the membrane attack complex (C5b-9) (24). Structural modifications, however, confer a prolonged duration of action to ravulizumab, allowing for an extended dosing interval of every 8 weeks compared to eculizumab. A comparative study of AEs for these two agents in myasthenia gravis (MG) suggests that while their mechanisms are similar, their clinical adverse event profiles are not identical. Eculizumab use is clearly associated with adverse events like Neisseria meningitidis bacteremia and septicemia, as documented in clinical studies and real-world settings (25–27). Our analysis identified 39 reports of Neisseria meningitidis infections associated with ravulizumab, indicating a similar risk profile for this specific, serious infection between the two drugs. This potentially fatal reaction underscores the critical importance of meningococcal vaccination prior to initiating therapy with either agent (28).

However, differences emerge for other AEs. Eculizumab treatment for MG has been associated with nasopharyngitis, gastric cancer, and embolic stroke. In contrast, ravulizumab therapy for MG has been linked to psoriatic arthropathy and peripheral vascular disease (29). Furthermore, the AE profile of these drugs varies significantly depending on the treated condition. Among the five AEs mentioned above (meningococcal infections, nasopharyngitis, gastric cancer, embolic stroke, psoriatic arthropathy, peripheral vascular disease), only nasopharyngitis has been reported in the context of treating PNH, aHUS, or NMOSD; the others have not been documented for these indications. This highlights that healthcare professionals must carefully consider the specific disease being treated and its associated AE profile when making therapeutic decisions.

Data extracted from the FAERS database indicate that fatigue is the most frequently reported AE associated with ravulizumab. However, its high reporting frequency may be attributed to several factors. Its characteristic clinical presentation facilitates accurate identification and documentation by healthcare providers, whereas concurrent non-drug-related symptoms may be incorrectly attributed to ravulizumab therapy. Anemia, identified as an AE not currently included in the drug label, may be linked to multiple factors. In patients with PNH, anemia could primarily result from bone marrow failure but may also arise from extravascular hemolysis (EVH) (30). EVH occurs due to C3b opsonization of PNH red blood cells, leading to their removal from circulation and subsequent destruction in the reticuloendothelial system. Furthermore, through sensitivity analysis, we mitigated the potential confounding effects of medications commonly used concomitantly with ravulizumab in standard clinical practice, such as prednisone, pyridostigmine, eculizumab, and intravenous immunoglobulin. Notably, pyridostigmine is a specific medication for MG treatment, and its frequent co-reporting reflects the greater utilization of ravulizumab in MG patients compared to other indications. This observation confirms that the characteristics of the data we extracted from FAERS align with real-world clinical practice.

Regarding the adverse reactions reported in published clinical studies, the incidence of adverse events associated with ravulizumab appears relatively high. In one randomized controlled trial (RCT) investigating ravulizumab for the treatment of MG, over 90% of participants reported treatment-emergent adverse events. Headache was reported in 23.1% of subjects, and diarrhea occurred in 17.2%. Concerning serious adverse events (SAEs), 16 SAEs were reported in 11 out of 169 participants, including pyelonephritis, pancreatitis, erysipelas, and Neisseria meningitidis bacteremia (31). Another RCT comparing ravulizumab and eculizumab for treating PNH similarly reported adverse events in 87.6% of patients. In the ravulizumab group, the most frequent AEs were headache (26.8%), nasopharyngitis (21.6%), and upper respiratory tract infection (18.6%) (32). The incidence of SAEs in this study was approximately 4%, which is lower than that observed in the MG trial. In a separate RCT evaluating ravulizumab for aHUS, the incidence of adverse events reached 100%. Headache, diarrhea, and vomiting were the most commonly reported AEs, while the SAE incidence was approximately 24% (33)—significantly higher than the rates observed in the MG and PNH trials. These findings suggest that the safety profile of ravulizumab may vary considerably across different indications. Therefore, during treatment, careful patient assessment tailored to the specific clinical context is essential to determine the most appropriate management strategy.

This study has several limitations that warrant consideration. First, as the FAERS database primarily relies on voluntary reporting by healthcare professionals and consumers, the potential for selection bias exists. Notably, in RCTs, physicians explicitly inform patients of potential AEs, enhancing reporting of even mild reactions, while real-world AEs are typically reported only when causing significant discomfort. This discrepancy may bias our results, as FAERS (a real-world database) may underreport mild AEs compared to RCTs. Second, the database lacks standardized grading for the severity of AEs and does not systematically differentiate between drug-induced AEs and those attributable to underlying comorbidities or concomitant medications. Although such differentiation is inherently challenging, this limitation complicates the accurate assessment of causality and severity, as disease-related symptoms may be reported as AEs. Consequently, clinical judgment remains essential for appropriate evaluation. Third, FAERS data reflect point-in-time reports following treatment and lack longitudinal follow-up information, which restricts the ability to assess long-term drug safety in real-world settings. Furthermore, the database exhibits significant geographic bias, with the majority of case reports originating from the United States, thereby limiting the generalizability of the findings to global populations and diverse clinical contexts. These inherent limitations underscore the necessity for complementary prospective studies to validate the pharmacovigilance signals identified in this analysis.

## 5 Conclusions

This pharmacovigilance study utilized the FAERS database to evaluate the real-world safety profile of ravulizumab. The analysis confirmed that frequently reported AEs, such as fatigue, asthenia, headache, malaise, dyspnea, back pain, feeling abnormal, and diplopia, were consistent with those described in the drug label. Importantly, several clinically significant AEs not currently listed in the prescribing information were identified, including anemia, dysphagia, urinary tract infection, and somnolence. These findings enhance healthcare professionals’ awareness of potential ravulizumab-associated toxicities, enabling more comprehensive risk assessment and informed management of potential adverse reactions when making treatment decisions for patients.

## Data Availability

The datasets presented in this study can be found in online repositories. The names of the repository/repositories and accession number(s) can be found below: https://fis.fda.gov/extensions/FPD-QDE-FAERS/FPD-QDE-FAERS.html.

## References

[1] Howard JF Jr . Myasthenia gravis: the role of complement at the neuromuscular junction. Ann N Y Acad Sci. (2018) 1412:113–28. doi: 10.1111/nyas.13522, PMID: 29266249

[2] Martinez Salazar A Mokhtari S Peguero E Jaffer M . The role of complement in the pathogenesis and treatment of myasthenia gravis. Cells. (2025) 14:739. doi: 10.3390/cells14100739, PMID: 40422242 PMC12110157

[3] Mantegazza R Vanoli F Frangiamore R Cavalcante P . Complement inhibition for the treatment of myasthenia gravis. Immunotargets Ther. (2020) 9:317–31. doi: 10.2147/ITT.S261414, PMID: 33365280 PMC7751298

[4] Morgan BP Harris CL . Complement therapeutics: history and current progress. Mol Immunol. (2003) 40:159–70. doi: 10.1016/S0161-5890(03)00111-1, PMID: 12914822

[5] Mizuno M Morgan BP . The possibilities and pitfalls for anti-complement therapies in inflammatory diseases. Curr Drug Targets Inflammation Allergy. (2004) 3:87–96. doi: 10.2174/1568010043483890, PMID: 15032645

[6] Kelly RJ Holt M Szer J . Pharmacological therapies in paroxysmal nocturnal haemoglobinuria: focus on complement inhibition. Drugs. (2025) 85:1413–28. doi: 10.1007/s40265-025-02235-4, PMID: 40986193 PMC12572029

[7] Trouw LA Pickering MC Blom AM . The complement system as a potential therapeutic target in rheumatic disease. Nat Rev Rheumatol. (2017) 13:538–47. doi: 10.1038/nrrheum.2017.125, PMID: 28794515

[8] Padilla Kelley T King H Malhotra A DeLoughery TG Martens K Shatzel JJ . Advancements in complement inhibition for PNH and primary complement-mediated thrombotic microangiopathy. Blood Adv. (2025) 9:3937–45. doi: 10.1182/bloodadvances.2024015777, PMID: 40354320 PMC12336697

[9] Sheridan D Yu ZX Zhang Y Patel R Sun F Lasaro MA . Design and preclinical characterization of ALXN1210: A novel anti-C5 antibody with extended duration of action. PloS One. (2018) 13:e0195909. doi: 10.1371/journal.pone.0195909, PMID: 29649283 PMC5897016

[10] Gilhus NE Tzartos S Evoli A Palace J Burns TM Verschuuren JJGM . Myasthenia gravis. Nat Rev Dis Primers. (2019) 5:30. doi: 10.1038/s41572-019-0079-y, PMID: 31048702

[11] Vu T Meisel A Mantegazza R Annane D Katsuno M Aguzzi R . Terminal complement inhibitor ravulizumab in generalized myasthenia gravis. NEJM Evid. (2022) 1:EVIDoa2100066. doi: 10.1056/EVIDoa2100066, PMID: 38319212

[12] Goodship TH Cook HT Fakhouri F Fervenza FC Frémeaux-Bacchi V Kavanagh D . Atypical hemolytic uremic syndrome and C3 glomerulopathy: conclusions from a “Kidney Disease: Improving Global Outcomes” (KDIGO) Controversies Conference. Kidney Int. (2017) 91:539–51. doi: 10.1016/j.kint.2016.10.005, PMID: 27989322

[13] Fakhouri F Zuber J Frémeaux-Bacchi V Loirat C . Haemolytic uraemic syndrome. Lancet. (2017) 390:681–96. doi: 10.1016/S0140-6736(17)30062-4, PMID: 28242109

[14] Raina R Krishnappa V Blaha T Kann T Hein W Burke L . Atypical hemolytic-uremic syndrome: an update on pathophysiology, diagnosis, and treatment. Ther Apher Dial. (2019) 23:4–21. doi: 10.1111/1744-9987.12763, PMID: 30294946

[15] Dixon BP Kavanagh D Aris ADM Adams B Kang HG Wang E . Ravulizumab in atypical hemolytic uremic syndrome: an analysis of 2-year efficacy and safety outcomes in 2 phase 3 trials. Kidney Med. (2024) 6:100855. doi: 10.1016/j.xkme.2024.100855, PMID: 39105067 PMC11298908

[16] Kumar . Signal Analysis in Pharmacovigilance Principles and Processes. Boca Raton: CRC Press (2024).

[17] Brown EG . Using MedDRA: implications for risk management. Drug Saf. (2004) 27:591–602. doi: 10.2165/00002018-200427080-00010, PMID: 15154830

[18] Rothman KJ Lanes S Sacks ST . The reporting odds ratio and its advantages over the proportional reporting ratio. Pharmacoepidemiol Drug Saf. (2004) 13:519–23. doi: 10.1002/pds.v13:8, PMID: 15317031

[19] Sharma A Kumar A . Identification of novel signal of clobazam-associated drug reaction with eosinophilia and systemic symptoms syndrome: A disproportionality analysis. Acta Neurol Scand. (2022) 146:623–7. doi: 10.1111/ane.v146.5, PMID: 36029138

[20] Javed F Kumar A . Identification of signal of clindamycin associated renal failure acute: A disproportionality analysis. Curr Drug Saf. (2024) 19:123–8. doi: 10.2174/1574886318666230228142856, PMID: 36852785

[21] Evans SJ Waller PC Davis S . Use of proportional reporting ratios (PRRs) for signal generation from spontaneous adverse drug reaction reports. Pharmacoepidemiol Drug Saf. (2001) 10:483–6. doi: 10.1002/pds.v10:6, PMID: 11828828

[22] Rivkees SA Szarfman A . Dissimilar hepatotoxicity profiles of propylthiouracil and methimazole in children. J Clin Endocrinol Metab. (2010) 95:3260–7. doi: 10.1210/jc.2009-2546, PMID: 20427502

[23] Ang PS Chen Z Chan CL Tai BC . Data mining spontaneous adverse drug event reports for safety signals in Singapore - a comparison of three different disproportionality measures. Expert Opin Drug Saf. (2016) 15:583–90. doi: 10.1517/14740338.2016.1167184, PMID: 26996192

[24] Vieira GD Boldrini VO Mader S Kümpfel T Meinl E Damasceno A . Ravulizumab and other complement inhibitors for the treatment of autoimmune disorders. Mult Scler Relat Disord. (2025) 95:106311. doi: 10.1016/j.msard.2025.106311, PMID: 39983521

[25] Dmytrijuk A Robie-Suh K Cohen MH Rieves D Weiss K Pazdur R . FDA report: eculizumab (Soliris) for the treatment of patients with paroxysmal nocturnal hemoglobinuria. Oncologist. (2008) 13:993–1000. doi: 10.1634/theoncologist, PMID: 18784156

[26] de Latour RP Szer J Weitz IC Röth A Höchsmann B Panse J . Pegcetacoplan versus eculizumab in patients with paroxysmal nocturnal haemoglobinuria (PEGASUS): 48-week follow-up of a randomised, open-label, phase 3, active-comparator, controlled trial. Lancet Haematol. (2022) 9:e648–59. doi: 10.1016/S2352-3026(22)00210-1, PMID: 36055332

[27] Nishimura JI Kawaguchi T Ito S Murai H Shimono A Matsuda T . Real-world safety profile of eculizumab in patients with paroxysmal nocturnal hemoglobinuria, atypical hemolytic uremic syndrome, or generalized myasthenia gravis: an integrated analysis of post-marketing surveillance in Japan. Int J Hematol. (2023) 118:419–31. doi: 10.1007/s12185-023-03630-x, PMID: 37515657

[28] Kelly RJ Holt M Vidler J Arnold LM Large J Forrest B . Treatment outcomes of complement protein C5 inhibition in 509 UK patients with paroxysmal nocturnal hemoglobinuria. Blood. (2024) 21:1157–66. doi: 10.1182/blood.2023021762, PMID: 38142401

[29] Wang L Chen J Li H Wang L Liu F Jiang X . Safety profile of complement C5 inhibitors and FcRn inhibitors in the treatment of myasthenia gravis: analysis of the FAERS database and disease-gene interaction network. Front Immunol. (2025) 16:1667249. doi: 10.3389/fimmu.2025.1667249, PMID: 41132654 PMC12540168

[30] Risitano AM Notaro R Marando L Serio B Ranaldi D Seneca E . Complement fraction 3 binding on erythrocytes as additional mechanism of disease in paroxysmal nocturnal hemoglobinuria patients treated by eculizumab. Blood. (2009) 113:4094–100. doi: 10.1182/blood-2008-11-189944, PMID: 19179465

[31] Vu TH Mantegazza R Annane D Katsuno M Meisel A Nicolle MW . Long-term efficacy and safety of ravulizumab in adults with anti-acetylcholine receptor antibody-positive generalized myasthenia gravis: final results from the phase 3 CHAMPION MG open-label extension. Eur J Neurol. (2025) 32:e70158. doi: 10.1111/ene.70158, PMID: 40241307 PMC12003558

[32] Kulasekararaj AG Hill A Rottinghaus ST Langemeijer S Wells R Gonzalez-Fernandez FA . Ravulizumab (ALXN1210) vs eculizumab in C5-inhibitor-experienced adult patients with PNH: the 302 study. Blood. (2019) 133:540–9. doi: 10.1182/blood-2018-09-876805, PMID: 30510079 PMC6368201

[33] Rondeau E Scully M Ariceta G Barbour T Cataland S Heyne N . The long-acting C5 inhibitor, Ravulizumab, is effective and safe in adult patients with atypical hemolytic uremic syndrome naïve to complement inhibitor treatment. Kidney Int. (2020) 97:1287–96. doi: 10.1016/j.kint.2020.01.035, PMID: 32299680

