# Supplementary material for: Analysis of adverse drug reactions associated with ravulizumab: a retrospective pharmacovigilance study utilizing the FAERS database
Source: Front Immunol. 2026 Feb 25;17:1736692. doi: 10.3389/fimmu.2026.1736692 (PMC12976008; doi:10.3389/fimmu.2026.1736692)
Supplement: Supplementary file 1 [file Table1.docx]

Supplementary Material

Supplementary Tables

Supplementary Table 1:

Two-by-two contingency table for disproportionality analyses.

|  | Target AEs | Other AEs | Total |
| --- | --- | --- | --- |
|  | a | b | a+b |
| Other drugs | c | d | c+d |
| Total | a+c | b+d | a+b+c+d |

Abbreviation: AEs, adverse events; a, number of reports containing both the target drug and target adverse drug reaction; b, number of reports containing other adverse drug reaction of the target drug; c, number of reports containing the target adverse drug reaction of other drugs; d, number of reports containing other drugs and other adverse drug reactions.

Supplementary Table 2:

Four major algorithms used for signal detection.

| Algorithms | Equation | Criteria |
| --- | --- | --- |
| ROR | ROR=ad/b/c | lower limit of 95% CI>1, N≥3 |
|  | 95%CI=e^ln(ROR)±1.96(1/a+1/b+1/c+1/d)^0.5^ |  |
| PRR | PRR=a(c+d)/c/(a+b) | PRR≥2, χ^2^≥4, N≥3 |
|  | χ^2^=[(ad-bc)^2](a+b+c+d)/[(a+b)(c+d)(a+c)(b+d)] |  |
| BCPNN | IC=log_2_a(a+b+c+d)(a+c)(a+b) | IC025>0 |
|  | 95%CI= E(IC) ± 2V(IC)^0.5 |  |
| MGPS | EBGM=a(a+b+c+d)/(a+c)/(a+b) | EBGM05>2 |
|  | 95%CI=e^ln(EBGM)±1.96(1/a+1/b+1/c+1/d)^0.5^ |  |

Abbreviation: a, number of reports containing both the target drug and target adverse drug reaction; b, number of reports containing other adverse drug reaction of the target drug; c, number of reports containing the target adverse drug reaction of other drugs; d, number of reports containing other drugs and other adverse drug reactions. 95%CI, 95% confidence interval; N, the number of reports; χ2, chi-squared; IC, information component; IC025, the lower limit of 95% CI of the IC; E(IC), the IC expectations; V(IC), the variance of IC; EBGM, empirical Bayesian geometric mean; EBGM05, the lower limit of 95% CI of EBGM.

Supplementary Table 3:

Top 50 most frequent adverse events for Ravulizumab​ at the preferred term (PT) level in males from FAERS data

| PT | Case numbers | ROR(95%CI) | PRR(χ^2^) | EBGM(EBGM05) | IC(IC025) |
| --- | --- | --- | --- | --- | --- |
| fatigue* | 433 | 5.39 ( 4.89 - 5.94 ) | 5.13 ( 1451.21 ) | 5.12 ( 4.64 ) | 2.35 ( 2.2 ) |
| asthenia* | 261 | 6.31 ( 5.58 - 7.14 ) | 6.13 ( 1120.52 ) | 6.1 ( 5.39 ) | 2.61 ( 2.4 ) |
| drug ineffective* | 193 | 1.28 ( 1.11 - 1.47 ) | 1.27 ( 11.29 ) | 1.27 ( 1.1 ) | 0.34 ( 0.13 ) |
| headache* | 170 | 3.64 ( 3.13 - 4.24 ) | 3.58 ( 317.12 ) | 3.57 ( 3.07 ) | 1.84 ( 1.59 ) |
| muscular weakness* | 167 | 14.8 ( 12.69 - 17.27 ) | 14.49 ( 2077.85 ) | 14.34 ( 12.29 ) | 3.84 ( 3.51 ) |
| diplopia* | 134 | 52.65 ( 44.23 - 62.66 ) | 51.72 ( 6412.75 ) | 49.78 ( 41.83 ) | 5.64 ( 4.94 ) |
| dyspnoea* | 126 | 2.06 ( 1.72 - 2.45 ) | 2.04 ( 67.06 ) | 2.04 ( 1.71 ) | 1.03 ( 0.76 ) |
| feeling abnormal* | 106 | 5.31 ( 4.38 - 6.44 ) | 5.25 ( 364.28 ) | 5.23 ( 4.32 ) | 2.39 ( 2.05 ) |
| therapeutic response shortened* | 106 | 20.02 ( 16.5 - 24.29 ) | 19.75 ( 1860.16 ) | 19.47 ( 16.05 ) | 4.28 ( 3.77 ) |
| back pain* | 91 | 4.06 ( 3.3 - 4.99 ) | 4.02 ( 206.27 ) | 4.01 ( 3.26 ) | 2 ( 1.65 ) |
| malaise* | 89 | 2.27 ( 1.84 - 2.8 ) | 2.25 ( 62.38 ) | 2.25 ( 1.83 ) | 1.17 ( 0.85 ) |
| pain* | 86 | 1.12 ( 0.91 - 1.39 ) | 1.12 ( 1.17 ) | 1.12 ( 0.91 ) | 0.17 ( -0.15 ) |
| haemoglobin decreased* | 85 | 6.99 ( 5.64 - 8.66 ) | 6.92 ( 429.14 ) | 6.89 ( 5.56 ) | 2.78 ( 2.38 ) |
| eyelid ptosis* | 82 | 102.55 ( 81.82 - 128.54 ) | 101.43 ( 7566.33 ) | 94.18 ( 75.14 ) | 6.56 ( 5.14 ) |
| diarrhoea | 80 | 1.06 ( 0.85 - 1.32 ) | 1.06 ( 0.27 ) | 1.06 ( 0.85 ) | 0.08 ( -0.24 ) |
| dysphagia* | 80 | 7.34 ( 5.88 - 9.15 ) | 7.27 ( 430.91 ) | 7.24 ( 5.8 ) | 2.86 ( 2.42 ) |
| haemolysis* | 75 | 82.51 ( 65.27 - 104.31 ) | 81.69 ( 5625.81 ) | 76.93 ( 60.85 ) | 6.27 ( 4.92 ) |
| arthralgia* | 73 | 1.8 ( 1.43 - 2.27 ) | 1.8 ( 25.91 ) | 1.8 ( 1.43 ) | 0.84 ( 0.49 ) |
| covid-19* | 72 | 1.71 ( 1.35 - 2.15 ) | 1.7 ( 20.87 ) | 1.7 ( 1.35 ) | 0.77 ( 0.41 ) |
| gait disturbance* | 68 | 3.4 ( 2.68 - 4.32 ) | 3.38 ( 113.77 ) | 3.37 ( 2.65 ) | 1.75 ( 1.35 ) |
| dizziness* | 68 | 1.46 ( 1.15 - 1.86 ) | 1.46 ( 9.8 ) | 1.46 ( 1.15 ) | 0.54 ( 0.18 ) |
| pain in extremity* | 68 | 2.68 ( 2.11 - 3.4 ) | 2.66 ( 70.72 ) | 2.66 ( 2.09 ) | 1.41 ( 1.03 ) |
| off label use | 63 | 0.44 ( 0.35 - 0.57 ) | 0.45 ( 43.79 ) | 0.45 ( 0.35 ) | -1.16 ( -1.51 ) |
| myasthenia gravis* | 59 | 45.66 ( 35.18 - 59.25 ) | 45.3 ( 2470.66 ) | 43.81 ( 33.76 ) | 5.45 ( 4.3 ) |
| pyrexia | 55 | 1.27 ( 0.98 - 1.66 ) | 1.27 ( 3.19 ) | 1.27 ( 0.97 ) | 0.35 ( -0.05 ) |
| symptom recurrence* | 55 | 49.68 ( 37.91 - 65.09 ) | 49.32 ( 2509 ) | 47.56 ( 36.3 ) | 5.57 ( 4.3 ) |
| nausea | 54 | 0.9 ( 0.69 - 1.17 ) | 0.9 ( 0.65 ) | 0.9 ( 0.69 ) | -0.16 ( -0.55 ) |
| death | 52 | 0.35 ( 0.27 - 0.47 ) | 0.36 ( 60.73 ) | 0.36 ( 0.27 ) | -1.48 ( -1.86 ) |
| muscle spasms* | 52 | 3.24 ( 2.46 - 4.26 ) | 3.22 ( 79.73 ) | 3.22 ( 2.45 ) | 1.69 ( 1.23 ) |
| weight decreased* | 52 | 1.41 ( 1.07 - 1.85 ) | 1.41 ( 6.2 ) | 1.41 ( 1.07 ) | 0.49 ( 0.08 ) |
| nasopharyngitis* | 50 | 2.74 ( 2.08 - 3.63 ) | 2.73 ( 54.94 ) | 2.73 ( 2.07 ) | 1.45 ( 0.99 ) |
| balance disorder* | 47 | 5.24 ( 3.93 - 6.99 ) | 5.22 ( 159.73 ) | 5.2 ( 3.9 ) | 2.38 ( 1.84 ) |
| condition aggravated | 46 | 1.14 ( 0.85 - 1.53 ) | 1.14 ( 0.81 ) | 1.14 ( 0.85 ) | 0.19 ( -0.24 ) |
| illness* | 44 | 2.93 ( 2.17 - 3.94 ) | 2.91 ( 55.3 ) | 2.91 ( 2.16 ) | 1.54 ( 1.05 ) |
| vision blurred* | 43 | 3.56 ( 2.64 - 4.81 ) | 3.55 ( 78.5 ) | 3.54 ( 2.62 ) | 1.82 ( 1.31 ) |
| somnolence* | 42 | 1.98 ( 1.46 - 2.69 ) | 1.98 ( 20.3 ) | 1.98 ( 1.46 ) | 0.98 ( 0.51 ) |
| myalgia* | 41 | 2.56 ( 1.89 - 3.49 ) | 2.55 ( 38.79 ) | 2.55 ( 1.88 ) | 1.35 ( 0.85 ) |
| speech disorder* | 41 | 7.05 ( 5.18 - 9.59 ) | 7.01 ( 210.49 ) | 6.98 ( 5.13 ) | 2.8 ( 2.16 ) |
| fall | 40 | 1.09 ( 0.8 - 1.49 ) | 1.09 ( 0.33 ) | 1.09 ( 0.8 ) | 0.13 ( -0.33 ) |
| cough | 40 | 1.21 ( 0.89 - 1.65 ) | 1.21 ( 1.44 ) | 1.21 ( 0.89 ) | 0.27 ( -0.19 ) |
| anaemia* | 39 | 1.83 ( 1.34 - 2.51 ) | 1.82 ( 14.56 ) | 1.82 ( 1.33 ) | 0.87 ( 0.38 ) |
| neck pain* | 36 | 7.3 ( 5.26 - 10.13 ) | 7.27 ( 193.64 ) | 7.23 ( 5.21 ) | 2.85 ( 2.15 ) |
| visual impairment* | 36 | 2.58 ( 1.86 - 3.57 ) | 2.57 ( 34.47 ) | 2.57 ( 1.85 ) | 1.36 ( 0.82 ) |
| general physical health deterioration* | 36 | 2.23 ( 1.61 - 3.09 ) | 2.22 ( 24.24 ) | 2.22 ( 1.6 ) | 1.15 ( 0.63 ) |
| infection* | 31 | 1.88 ( 1.32 - 2.68 ) | 1.88 ( 12.75 ) | 1.88 ( 1.32 ) | 0.91 ( 0.36 ) |
| blood lactate dehydrogenase increased* | 30 | 18.77 ( 13.08 - 26.93 ) | 18.7 ( 495.49 ) | 18.45 ( 12.85 ) | 4.21 ( 3.04 ) |
| weight increased | 29 | 1.34 ( 0.93 - 1.93 ) | 1.34 ( 2.48 ) | 1.34 ( 0.93 ) | 0.42 ( -0.12 ) |
| poor venous access* | 29 | 22.01 ( 15.24 - 31.8 ) | 21.93 ( 569.83 ) | 21.58 ( 14.94 ) | 4.43 ( 3.15 ) |
| abdominal discomfort* | 29 | 1.72 ( 1.2 - 2.48 ) | 1.72 ( 8.77 ) | 1.72 ( 1.19 ) | 0.78 ( 0.22 ) |
| blood pressure increased* | 29 | 1.64 ( 1.14 - 2.36 ) | 1.63 ( 7.13 ) | 1.63 ( 1.13 ) | 0.71 ( 0.15 ) |

Abbreviation: Asterisks (*) indicate statistically significant signals in algorithm; ROR, reporting odds ratio; PRR, proportional reporting ratio; EBGM, empirical Bayesian geometric mean; EBGM05, the lower limit of the 95% CI of EBGM; IC, information component; IC025, the lower limit of the 95% CI of the IC; CI, confidence interval; PT,preferred term; AEs, adverse events.

Supplementary Table 4:

Top 50 most frequent adverse events for Ravulizumab in at the PT level in females from FAERS data

| PT | Case numbers | ROR(95%CI) | PRR(χ^2^) | EBGM(EBGM05) | IC(IC025) |
| --- | --- | --- | --- | --- | --- |
| fatigue* | 535 | 5 ( 4.58 - 5.46 ) | 4.75 ( 1599.37 ) | 4.74 ( 4.34 ) | 2.24 ( 2.1 ) |
| headache* | 287 | 3.17 ( 2.81 - 3.56 ) | 3.09 ( 410.13 ) | 3.09 ( 2.75 ) | 1.63 ( 1.44 ) |
| asthenia* | 202 | 4.49 ( 3.9 - 5.16 ) | 4.41 ( 533.43 ) | 4.4 ( 3.82 ) | 2.14 ( 1.91 ) |
| drug ineffective | 157 | 0.86 ( 0.73 - 1 ) | 0.86 ( 3.75 ) | 0.86 ( 0.73 ) | -0.22 ( -0.45 ) |
| nausea | 131 | 1.16 ( 0.97 - 1.38 ) | 1.16 ( 2.8 ) | 1.16 ( 0.97 ) | 0.21 ( -0.05 ) |
| malaise* | 124 | 2.08 ( 1.74 - 2.49 ) | 2.07 ( 68.7 ) | 2.07 ( 1.73 ) | 1.05 ( 0.77 ) |
| back pain** | 124 | 3.98 ( 3.34 - 4.76 ) | 3.94 ( 272.31 ) | 3.93 ( 3.29 ) | 1.98 ( 1.68 ) |
| dyspnoea* | 118 | 1.63 ( 1.36 - 1.95 ) | 1.62 ( 28.19 ) | 1.62 ( 1.35 ) | 0.7 ( 0.42 ) |
| muscular weakness* | 116 | 8.58 ( 7.14 - 10.32 ) | 8.48 ( 763.07 ) | 8.45 ( 7.03 ) | 3.08 ( 2.72 ) |
| haemoglobin decreased* | 107 | 9.91 ( 8.18 - 11.99 ) | 9.79 ( 841.48 ) | 9.75 ( 8.05 ) | 3.28 ( 2.89 ) |
| pain | 102 | 1.04 ( 0.85 - 1.26 ) | 1.04 ( 0.14 ) | 1.04 ( 0.85 ) | 0.05 ( -0.23 ) |
| diarrhoea | 99 | 1.07 ( 0.88 - 1.31 ) | 1.07 ( 0.47 ) | 1.07 ( 0.88 ) | 0.1 ( -0.19 ) |
| feeling abnormal* | 92 | 2.92 ( 2.38 - 3.59 ) | 2.9 ( 115.08 ) | 2.9 ( 2.36 ) | 1.54 ( 1.21 ) |
| dizziness* | 87 | 1.38 ( 1.12 - 1.7 ) | 1.37 ( 8.92 ) | 1.37 ( 1.11 ) | 0.46 ( 0.14 ) |
| arthralgia* | 83 | 1.26 ( 1.01 - 1.56 ) | 1.25 ( 4.33 ) | 1.25 ( 1.01 ) | 0.33 ( 0.01 ) |
| off label use | 78 | 0.57 ( 0.46 - 0.72 ) | 0.58 ( 24.7 ) | 0.58 ( 0.46 ) | -0.79 ( -1.11 ) |
| pain in extremity* | 74 | 1.81 ( 1.44 - 2.28 ) | 1.8 ( 26.57 ) | 1.8 ( 1.43 ) | 0.85 ( 0.5 ) |
| diplopia* | 70 | 24.72 ( 19.51 - 31.33 ) | 24.52 ( 1560.08 ) | 24.23 ( 19.12 ) | 4.6 ( 3.84 ) |
| myasthenia gravis* | 68 | 79.88 ( 62.62 - 101.9 ) | 79.23 ( 5045.61 ) | 76.14 ( 59.69 ) | 6.25 ( 4.83 ) |
| gait disturbance* | 64 | 2.48 ( 1.94 - 3.17 ) | 2.47 ( 55.89 ) | 2.46 ( 1.93 ) | 1.3 ( 0.91 ) |
| nasopharyngitis* | 64 | 2.09 ( 1.63 - 2.67 ) | 2.08 ( 36.11 ) | 2.08 ( 1.63 ) | 1.06 ( 0.67 ) |
| eyelid ptosis* | 63 | 52.04 ( 40.48 - 66.9 ) | 51.66 ( 3048.26 ) | 50.33 ( 39.15 ) | 5.65 ( 4.46 ) |
| therapeutic response shortened* | 63 | 10 ( 7.8 - 12.82 ) | 9.93 ( 503.6 ) | 9.88 ( 7.71 ) | 3.3 ( 2.75 ) |
| symptom recurrence* | 62 | 49.22 ( 38.22 - 63.39 ) | 48.86 ( 2835.12 ) | 47.68 ( 37.02 ) | 5.58 ( 4.41 ) |
| illness* | 61 | 2.22 ( 1.73 - 2.86 ) | 2.21 ( 40.59 ) | 2.21 ( 1.72 ) | 1.14 ( 0.75 ) |
| fall* | 60 | 1.36 ( 1.06 - 1.76 ) | 1.36 ( 5.79 ) | 1.36 ( 1.06 ) | 0.44 ( 0.07 ) |
| covid-19* | 59 | 1.2 ( 0.93 - 1.55 ) | 1.19 ( 1.88 ) | 1.19 ( 0.92 ) | 0.26 ( -0.12 ) |
| pyrexia* | 58 | 1.41 ( 1.09 - 1.83 ) | 1.41 ( 6.98 ) | 1.41 ( 1.09 ) | 0.5 ( 0.11 ) |
| dysphagia* | 58 | 5.54 ( 4.28 - 7.18 ) | 5.51 ( 213.9 ) | 5.5 ( 4.25 ) | 2.46 ( 1.98 ) |
| cough* | 57 | 1.35 ( 1.04 - 1.75 ) | 1.35 ( 5.08 ) | 1.35 ( 1.04 ) | 0.43 ( 0.04 ) |
| haemolysis* | 57 | 99.22 ( 75.97 - 129.6 ) | 98.55 ( 5236.34 ) | 93.8 ( 71.82 ) | 6.55 ( 4.78 ) |
| condition aggravated | 54 | 1.15 ( 0.88 - 1.51 ) | 1.15 ( 1.1 ) | 1.15 ( 0.88 ) | 0.21 ( -0.19 ) |
| somnolence* | 51 | 2.17 ( 1.65 - 2.86 ) | 2.17 ( 32.12 ) | 2.17 ( 1.64 ) | 1.12 ( 0.68 ) |
| urinary tract infection* | 51 | 1.75 ( 1.33 - 2.3 ) | 1.75 ( 16.27 ) | 1.74 ( 1.32 ) | 0.8 ( 0.38 ) |
| exposure during pregnancy* | 51 | 3.58 ( 2.71 - 4.71 ) | 3.56 ( 93.88 ) | 3.56 ( 2.7 ) | 1.83 ( 1.36 ) |
| infusion related reaction* | 50 | 4.47 ( 3.39 - 5.91 ) | 4.45 ( 133.76 ) | 4.45 ( 3.37 ) | 2.15 ( 1.65 ) |
| myalgia* | 49 | 2.49 ( 1.88 - 3.3 ) | 2.48 ( 43.31 ) | 2.48 ( 1.87 ) | 1.31 ( 0.86 ) |
| blood pressure increased* | 48 | 2.39 ( 1.8 - 3.17 ) | 2.38 ( 38.43 ) | 2.38 ( 1.79 ) | 1.25 ( 0.8 ) |
| anaemia* | 43 | 2.1 ( 1.55 - 2.83 ) | 2.09 ( 24.57 ) | 2.09 ( 1.55 ) | 1.06 ( 0.59 ) |
| anxiety | 43 | 1.18 ( 0.87 - 1.59 ) | 1.18 ( 1.15 ) | 1.18 ( 0.87 ) | 0.23 ( -0.21 ) |
| vomiting | 41 | 0.64 ( 0.47 - 0.87 ) | 0.64 ( 8.19 ) | 0.64 ( 0.47 ) | -0.64 ( -1.07 ) |
| stress* | 40 | 3.23 ( 2.36 - 4.4 ) | 3.21 ( 61.01 ) | 3.21 ( 2.35 ) | 1.68 ( 1.15 ) |
| infection* | 40 | 1.93 ( 1.41 - 2.63 ) | 1.92 ( 17.74 ) | 1.92 ( 1.41 ) | 0.94 ( 0.46 ) |
| muscle spasms* | 39 | 1.68 ( 1.22 - 2.3 ) | 1.67 ( 10.63 ) | 1.67 ( 1.22 ) | 0.74 ( 0.26 ) |
| pruritus | 38 | 0.63 ( 0.46 - 0.86 ) | 0.63 ( 8.3 ) | 0.63 ( 0.46 ) | -0.67 ( -1.12 ) |
| rash | 37 | 0.57 ( 0.41 - 0.79 ) | 0.57 ( 12.01 ) | 0.57 ( 0.41 ) | -0.81 ( -1.26 ) |
| abdominal discomfort | 37 | 1.37 ( 0.99 - 1.89 ) | 1.37 ( 3.66 ) | 1.37 ( 0.99 ) | 0.45 ( -0.03 ) |
| chest pain* | 35 | 1.71 ( 1.23 - 2.38 ) | 1.71 ( 10.26 ) | 1.71 ( 1.22 ) | 0.77 ( 0.26 ) |
| poor venous access* | 35 | 21.59 ( 15.46 - 30.15 ) | 21.5 ( 676.8 ) | 21.28 ( 15.24 ) | 4.41 ( 3.28 ) |
| insomnia | 34 | 1.08 ( 0.77 - 1.51 ) | 1.08 ( 0.2 ) | 1.08 ( 0.77 ) | 0.11 ( -0.38 ) |

Abbreviation: Asterisks (*) indicate statistically significant signals in algorithm; ROR, reporting odds ratio; PRR, proportional reporting ratio; EBGM, empirical Bayesian geometric mean; EBGM05, the lower limit of the 95% CI of EBGM; IC, information component; IC025, the lower limit of the 95% CI of the IC; CI, confidence interval; PT,preferred term; AEs, adverse events.

Supplementary Table 5:

Top 50 most frequent adverse events at the PT level for Ravulizumab in patients aged under 18 from FAERS data

| PT | Case numbers | ROR(95%CI) | PRR(χ^2^) | EBGM(EBGM05) | IC(IC025) |
| --- | --- | --- | --- | --- | --- |
| haemolysis* | 13 | 161.49 ( 91.69 - 284.44 ) | 156.75 ( 1909.22 ) | 148.78 ( 84.47 ) | 7.22 ( 2.88 ) |
| off label use | 12 | 0.81 ( 0.46 - 1.44 ) | 0.82 ( 0.5 ) | 0.82 ( 0.46 ) | -0.29 ( -1.08 ) |
| infusion related reaction* | 9 | 17.74 ( 9.15 - 34.4 ) | 17.4 ( 138.46 ) | 17.3 ( 8.92 ) | 4.11 ( 1.79 ) |
| nausea | 6 | 2.11 ( 0.94 - 4.72 ) | 2.1 ( 3.45 ) | 2.09 ( 0.94 ) | 1.07 ( -0.25 ) |
| rash | 6 | 1.4 ( 0.62 - 3.13 ) | 1.39 ( 0.67 ) | 1.39 ( 0.62 ) | 0.48 ( -0.7 ) |
| urticaria* | 6 | 3.94 ( 1.76 - 8.81 ) | 3.9 ( 12.94 ) | 3.89 ( 1.74 ) | 1.96 ( 0.36 ) |
| drug ineffective | 6 | 0.57 ( 0.26 - 1.28 ) | 0.58 ( 1.89 ) | 0.58 ( 0.26 ) | -0.79 ( -1.8 ) |
| myasthenia gravis* | 6 | 166.74 ( 72.86 - 381.58 ) | 164.48 ( 922.79 ) | 155.72 ( 68.05 ) | 7.28 ( 1.62 ) |
| condition aggravated | 6 | 2.21 ( 0.98 - 4.94 ) | 2.19 ( 3.89 ) | 2.19 ( 0.98 ) | 1.13 ( -0.2 ) |
| dyspnoea* | 5 | 2.84 ( 1.18 - 6.86 ) | 2.82 ( 5.89 ) | 2.82 ( 1.17 ) | 1.49 ( -0.08 ) |
| pyrexia | 5 | 1.06 ( 0.44 - 2.56 ) | 1.06 ( 0.02 ) | 1.06 ( 0.44 ) | 0.08 ( -1.12 ) |
| death* | 5 | 2.74 ( 1.14 - 6.63 ) | 2.72 ( 5.48 ) | 2.72 ( 1.13 ) | 1.45 ( -0.11 ) |
| headache | 5 | 1.77 ( 0.73 - 4.28 ) | 1.76 ( 1.66 ) | 1.76 ( 0.73 ) | 0.82 ( -0.54 ) |
| anaphylactic reaction* | 5 | 7.58 ( 3.13 - 18.31 ) | 7.5 ( 28.14 ) | 7.48 ( 3.1 ) | 2.9 ( 0.66 ) |
| haemoglobin decreased* | 5 | 12.4 ( 5.13 - 30 ) | 12.27 ( 51.59 ) | 12.22 ( 5.05 ) | 3.61 ( 0.9 ) |
| pruritus | 4 | 1.13 ( 0.42 - 3.02 ) | 1.13 ( 0.06 ) | 1.13 ( 0.42 ) | 0.17 ( -1.17 ) |
| cough | 4 | 2.24 ( 0.84 - 6.01 ) | 2.23 ( 2.73 ) | 2.23 ( 0.83 ) | 1.16 ( -0.46 ) |
| hospitalisation* | 4 | 4.35 ( 1.63 - 11.66 ) | 4.32 ( 10.22 ) | 4.32 ( 1.61 ) | 2.11 ( 0.08 ) |
| nasopharyngitis* | 4 | 3.71 ( 1.38 - 9.93 ) | 3.68 ( 7.83 ) | 3.68 ( 1.37 ) | 1.88 ( -0.04 ) |
| disease recurrence* | 4 | 9.46 ( 3.53 - 25.35 ) | 9.38 ( 29.88 ) | 9.35 ( 3.49 ) | 3.23 ( 0.51 ) |
| pneumonia aspiration* | 4 | 16.24 ( 6.05 - 43.58 ) | 16.1 ( 56.36 ) | 16.02 ( 5.97 ) | 4 ( 0.7 ) |
| extravascular haemolysis* | 4 | 1955 ( 549.77 - 6952.11 ) | 1937.24 ( 4644.62 ) | 1162.74 ( 326.98 ) | 10.18 ( 0.75 ) |
| paroxysmal nocturnal haemoglobinuria* | 4 | 1675.72 ( 488.78 - 5744.93 ) | 1660.49 ( 4221.65 ) | 1057.04 ( 308.32 ) | 10.05 ( 0.77 ) |
| atypical haemolytic uraemic syndrome* | 4 | 120.92 ( 44.29 - 330.14 ) | 119.83 ( 452.72 ) | 115.12 ( 42.17 ) | 6.85 ( 0.94 ) |
| thrombotic microangiopathy* | 4 | 17.68 ( 6.59 - 47.47 ) | 17.53 ( 62.01 ) | 17.43 ( 6.49 ) | 4.12 ( 0.72 ) |
| anaemia* | 3 | 3.27 ( 1.05 - 10.2 ) | 3.26 ( 4.7 ) | 3.26 ( 1.05 ) | 1.7 ( -0.39 ) |
| pain | 3 | 1.97 ( 0.63 - 6.14 ) | 1.96 ( 1.42 ) | 1.96 ( 0.63 ) | 0.97 ( -0.79 ) |
| seizure | 3 | 1.1 ( 0.35 - 3.42 ) | 1.1 ( 0.03 ) | 1.1 ( 0.35 ) | 0.14 ( -1.35 ) |
| malaise | 3 | 1.91 ( 0.61 - 5.94 ) | 1.9 ( 1.29 ) | 1.9 ( 0.61 ) | 0.93 ( -0.82 ) |
| abdominal pain | 3 | 1.62 ( 0.52 - 5.04 ) | 1.61 ( 0.7 ) | 1.61 ( 0.52 ) | 0.69 ( -0.97 ) |
| renal impairment* | 3 | 4.31 ( 1.38 - 13.42 ) | 4.29 ( 7.56 ) | 4.28 ( 1.37 ) | 2.1 ( -0.22 ) |
| hypotension | 3 | 2.23 ( 0.71 - 6.93 ) | 2.22 ( 2.01 ) | 2.22 ( 0.71 ) | 1.15 ( -0.69 ) |
| dizziness | 3 | 2.27 ( 0.73 - 7.06 ) | 2.26 ( 2.11 ) | 2.26 ( 0.73 ) | 1.18 ( -0.67 ) |
| device related sepsis* | 3 | 69.11 ( 21.91 - 217.98 ) | 68.64 ( 195.38 ) | 67.08 ( 21.27 ) | 6.07 ( 0.47 ) |
| meningococcal infection* | 3 | 74.38 ( 23.56 - 234.84 ) | 73.88 ( 210.35 ) | 72.07 ( 22.83 ) | 6.17 ( 0.47 ) |
| acute respiratory distress syndrome* | 3 | 11.48 ( 3.68 - 35.82 ) | 11.41 ( 28.4 ) | 11.37 ( 3.64 ) | 3.51 ( 0.21 ) |
| blood lactate dehydrogenase increased* | 3 | 20.5 ( 6.56 - 64.07 ) | 20.37 ( 54.89 ) | 20.23 ( 6.47 ) | 4.34 ( 0.34 ) |
| flushing | 2 | 5.48 ( 1.36 - 22 ) | 5.46 ( 7.27 ) | 5.45 ( 1.36 ) | 2.45 ( -0.54 ) |
| delirium* | 2 | 8.31 ( 2.07 - 33.41 ) | 8.28 ( 12.77 ) | 8.26 ( 2.05 ) | 3.05 ( -0.4 ) |
| chills | 2 | 5.64 ( 1.4 - 22.66 ) | 5.62 ( 7.59 ) | 5.61 ( 1.4 ) | 2.49 ( -0.53 ) |
| vomiting | 2 | 0.44 ( 0.11 - 1.77 ) | 0.44 ( 1.41 ) | 0.44 ( 0.11 ) | -1.17 ( -2.55 ) |
| anxiety | 2 | 1.52 ( 0.38 - 6.12 ) | 1.52 ( 0.36 ) | 1.52 ( 0.38 ) | 0.61 ( -1.3 ) |
| cholelithiasis* | 2 | 19.52 ( 4.84 - 78.67 ) | 19.44 ( 34.75 ) | 19.31 ( 4.79 ) | 4.27 ( -0.24 ) |
| hypertension | 2 | 2.12 ( 0.53 - 8.52 ) | 2.12 ( 1.18 ) | 2.12 ( 0.53 ) | 1.08 ( -1.05 ) |
| hypervolaemia* | 2 | 25.6 ( 6.34 - 103.32 ) | 25.49 ( 46.66 ) | 25.28 ( 6.26 ) | 4.66 ( -0.21 ) |
| chest pain | 2 | 3.9 ( 0.97 - 15.65 ) | 3.88 ( 4.28 ) | 3.88 ( 0.97 ) | 1.96 ( -0.69 ) |
| polyserositis* | 2 | 486.52 ( 108.57 - 2180.21 ) | 484.31 ( 826.83 ) | 415.27 ( 92.67 ) | 8.7 ( -0.25 ) |
| back pain | 2 | 4.16 ( 1.04 - 16.7 ) | 4.15 ( 4.77 ) | 4.14 ( 1.03 ) | 2.05 ( -0.66 ) |
| proteinuria | 2 | 7.12 ( 1.77 - 28.59 ) | 7.09 ( 10.44 ) | 7.07 ( 1.76 ) | 2.82 ( -0.45 ) |
| haemothorax* | 2 | 61.45 ( 15.1 - 250.09 ) | 61.18 ( 115.95 ) | 59.94 ( 14.73 ) | 5.91 ( -0.16 ) |

Abbreviation: Asterisks (*) indicate statistically significant signals in algorithm; ROR, reporting odds ratio; PRR, proportional reporting ratio; EBGM, empirical Bayesian geometric mean; EBGM05, the lower limit of the 95% CI of EBGM; IC, information component; IC025, the lower limit of the 95% CI of the IC; CI, confidence interval; PT, preferred term.

Supplementary Table 6:

Top 50 most frequent adverse events for Ravulizumab at the PT level in patients aged 18 to 65 from FAERS data

| PT | Case numbers | ROR(95%CI) | PRR(χ^2^) | EBGM(EBGM05) | IC(IC025) |
| --- | --- | --- | --- | --- | --- |
| fatigue* | 132 | 3.43 ( 2.88 - 4.08 ) | 3.33 ( 217.18 ) | 3.32 ( 2.79 ) | 1.73 ( 1.45 ) |
| headache* | 86 | 2.53 ( 2.04 - 3.14 ) | 2.49 ( 77.35 ) | 2.49 ( 2.01 ) | 1.31 ( 0.98 ) |
| myasthenia gravis* | 56 | 194.67 ( 148.38 - 255.41 ) | 191.21 ( 10027.68 ) | 180.99 ( 137.95 ) | 7.5 ( 5.05 ) |
| pyrexia* | 53 | 2.85 ( 2.17 - 3.74 ) | 2.82 ( 62.49 ) | 2.82 ( 2.15 ) | 1.49 ( 1.05 ) |
| nausea | 45 | 1.1 ( 0.82 - 1.48 ) | 1.1 ( 0.41 ) | 1.1 ( 0.82 ) | 0.14 ( -0.3 ) |
| haemolysis* | 45 | 136.86 ( 101.38 - 184.75 ) | 134.91 ( 5751.38 ) | 129.75 ( 96.11 ) | 7.02 ( 4.66 ) |
| off label use | 40 | 0.71 ( 0.52 - 0.96 ) | 0.71 ( 4.84 ) | 0.71 ( 0.52 ) | -0.49 ( -0.94 ) |
| covid-19* | 39 | 1.91 ( 1.4 - 2.63 ) | 1.9 ( 16.82 ) | 1.9 ( 1.39 ) | 0.93 ( 0.44 ) |
| malaise* | 39 | 2.03 ( 1.48 - 2.78 ) | 2.02 ( 20.08 ) | 2.02 ( 1.47 ) | 1.01 ( 0.52 ) |
| haemoglobin decreased* | 38 | 9.62 ( 6.99 - 13.26 ) | 9.52 ( 289.32 ) | 9.5 ( 6.89 ) | 3.25 ( 2.5 ) |
| pain | 36 | 1.16 ( 0.84 - 1.62 ) | 1.16 ( 0.83 ) | 1.16 ( 0.84 ) | 0.22 ( -0.27 ) |
| drug ineffective | 36 | 0.62 ( 0.45 - 0.86 ) | 0.63 ( 8.2 ) | 0.63 ( 0.45 ) | -0.68 ( -1.14 ) |
| dyspnoea | 34 | 1.31 ( 0.94 - 1.84 ) | 1.31 ( 2.52 ) | 1.31 ( 0.93 ) | 0.39 ( -0.11 ) |
| asthenia* | 32 | 2.05 ( 1.44 - 2.9 ) | 2.04 ( 16.96 ) | 2.04 ( 1.44 ) | 1.03 ( 0.48 ) |
| back pain* | 30 | 2.7 ( 1.88 - 3.87 ) | 2.68 ( 31.76 ) | 2.68 ( 1.87 ) | 1.42 ( 0.83 ) |
| arthralgia | 26 | 1.06 ( 0.72 - 1.56 ) | 1.06 ( 0.1 ) | 1.06 ( 0.72 ) | 0.09 ( -0.47 ) |
| anaemia* | 25 | 3.58 ( 2.42 - 5.31 ) | 3.56 ( 46.1 ) | 3.56 ( 2.4 ) | 1.83 ( 1.13 ) |
| death | 25 | 1.12 ( 0.75 - 1.66 ) | 1.12 ( 0.31 ) | 1.12 ( 0.75 ) | 0.16 ( -0.42 ) |
| nasopharyngitis* | 25 | 2.22 ( 1.5 - 3.29 ) | 2.21 ( 16.61 ) | 2.21 ( 1.49 ) | 1.14 ( 0.51 ) |
| condition aggravated | 24 | 1.19 ( 0.8 - 1.78 ) | 1.19 ( 0.73 ) | 1.19 ( 0.8 ) | 0.25 ( -0.34 ) |
| myalgia* | 23 | 2.94 ( 1.95 - 4.44 ) | 2.93 ( 29.28 ) | 2.93 ( 1.94 ) | 1.55 ( 0.85 ) |
| cough* | 23 | 1.61 ( 1.07 - 2.43 ) | 1.61 ( 5.28 ) | 1.61 ( 1.07 ) | 0.68 ( 0.06 ) |
| diarrhoea | 23 | 0.75 ( 0.5 - 1.13 ) | 0.75 ( 1.91 ) | 0.75 ( 0.5 ) | -0.41 ( -0.99 ) |
| feeling abnormal* | 22 | 2.16 ( 1.42 - 3.29 ) | 2.16 ( 13.68 ) | 2.16 ( 1.42 ) | 1.11 ( 0.43 ) |
| blood lactate dehydrogenase increased* | 22 | 40.14 ( 26.33 - 61.2 ) | 39.86 ( 823.95 ) | 39.41 ( 25.85 ) | 5.3 ( 3.28 ) |
| vomiting | 21 | 0.9 ( 0.58 - 1.38 ) | 0.9 ( 0.25 ) | 0.9 ( 0.58 ) | -0.16 ( -0.77 ) |
| infection* | 21 | 2.77 ( 1.81 - 4.26 ) | 2.76 ( 23.65 ) | 2.76 ( 1.8 ) | 1.47 ( 0.74 ) |
| dizziness | 21 | 0.94 ( 0.61 - 1.44 ) | 0.94 ( 0.08 ) | 0.94 ( 0.61 ) | -0.09 ( -0.7 ) |
| platelet count decreased* | 21 | 4.9 ( 3.19 - 7.53 ) | 4.88 ( 64.7 ) | 4.87 ( 3.17 ) | 2.28 ( 1.43 ) |
| chest pain* | 20 | 2.28 ( 1.47 - 3.54 ) | 2.27 ( 14.3 ) | 2.27 ( 1.46 ) | 1.18 ( 0.47 ) |
| pain in extremity | 19 | 1.38 ( 0.88 - 2.16 ) | 1.37 ( 1.94 ) | 1.37 ( 0.87 ) | 0.46 ( -0.22 ) |
| blood pressure increased* | 19 | 2.55 ( 1.62 - 4 ) | 2.54 ( 17.78 ) | 2.54 ( 1.62 ) | 1.34 ( 0.59 ) |
| infusion related reaction* | 19 | 3.71 ( 2.36 - 5.82 ) | 3.69 ( 37.28 ) | 3.69 ( 2.35 ) | 1.88 ( 1.05 ) |
| chills* | 18 | 2.8 ( 1.76 - 4.46 ) | 2.79 ( 20.74 ) | 2.79 ( 1.76 ) | 1.48 ( 0.69 ) |
| sepsis* | 17 | 3.61 ( 2.24 - 5.82 ) | 3.6 ( 31.92 ) | 3.6 ( 2.23 ) | 1.85 ( 0.97 ) |
| rash | 17 | 0.74 ( 0.46 - 1.19 ) | 0.74 ( 1.56 ) | 0.74 ( 0.46 ) | -0.43 ( -1.1 ) |
| abdominal pain | 17 | 1.3 ( 0.81 - 2.1 ) | 1.3 ( 1.2 ) | 1.3 ( 0.81 ) | 0.38 ( -0.32 ) |
| pneumonia | 17 | 1.21 ( 0.75 - 1.94 ) | 1.2 ( 0.6 ) | 1.2 ( 0.75 ) | 0.27 ( -0.43 ) |
| sars-cov-2 test positive* | 16 | 9.32 ( 5.7 - 15.24 ) | 9.28 ( 117.91 ) | 9.26 ( 5.66 ) | 3.21 ( 1.94 ) |
| pruritus | 15 | 0.66 ( 0.4 - 1.1 ) | 0.66 ( 2.62 ) | 0.66 ( 0.4 ) | -0.6 ( -1.29 ) |
| fall | 15 | 1.51 ( 0.91 - 2.5 ) | 1.5 ( 2.54 ) | 1.5 ( 0.91 ) | 0.59 ( -0.18 ) |
| eyelid ptosis* | 15 | 37.42 ( 22.47 - 62.31 ) | 37.24 ( 523.32 ) | 36.85 ( 22.12 ) | 5.2 ( 2.78 ) |
| somnolence | 14 | 1.58 ( 0.94 - 2.68 ) | 1.58 ( 2.99 ) | 1.58 ( 0.93 ) | 0.66 ( -0.14 ) |
| diplopia* | 13 | 10.78 ( 6.24 - 18.6 ) | 10.74 ( 114.45 ) | 10.7 ( 6.2 ) | 3.42 ( 1.89 ) |
| illness | 13 | 1.71 ( 0.99 - 2.95 ) | 1.71 ( 3.83 ) | 1.71 ( 0.99 ) | 0.77 ( -0.07 ) |
| muscle spasms | 13 | 1.58 ( 0.92 - 2.72 ) | 1.58 ( 2.75 ) | 1.58 ( 0.91 ) | 0.66 ( -0.18 ) |
| muscular weakness* | 13 | 2.53 ( 1.47 - 4.37 ) | 2.53 ( 12.02 ) | 2.53 ( 1.47 ) | 1.34 ( 0.41 ) |
| blood creatinine increased* | 13 | 4.55 ( 2.64 - 7.85 ) | 4.54 ( 35.85 ) | 4.53 ( 2.63 ) | 2.18 ( 1.08 ) |
| erythema | 12 | 0.99 ( 0.56 - 1.74 ) | 0.99 ( 0 ) | 0.99 ( 0.56 ) | -0.02 ( -0.82 ) |
| thrombocytopenia* | 12 | 2.28 ( 1.29 - 4.01 ) | 2.27 ( 8.55 ) | 2.27 ( 1.29 ) | 1.18 ( 0.25 ) |

Abbreviation: Asterisks (*) indicate statistically significant signals in algorithm; ROR, reporting odds ratio; PRR, proportional reporting ratio; EBGM, empirical Bayesian geometric mean; EBGM05, the lower limit of the 95% CI of EBGM; IC, information component; IC025, the lower limit of the 95% CI of the IC; CI, confidence interval; PT, preferred term.

Supplementary Table 7:

Top 50 most frequent adverse events for Ravulizumab at the PT level in patients aged over 65 from FAERS data

| PT | Case numbers | ROR(95%CI) | PRR(χ^2^) | EBGM(EBGM05) | IC(IC025) |
| --- | --- | --- | --- | --- | --- |
| fatigue* | 112 | 3.56 ( 2.94 - 4.3 ) | 3.44 ( 196.32 ) | 3.44 ( 2.84 ) | 1.78 ( 1.47 ) |
| asthenia* | 67 | 3.47 ( 2.72 - 4.42 ) | 3.4 ( 114.23 ) | 3.4 ( 2.66 ) | 1.76 ( 1.36 ) |
| death | 53 | 0.96 ( 0.73 - 1.26 ) | 0.96 ( 0.11 ) | 0.96 ( 0.73 ) | -0.06 ( -0.46 ) |
| drug ineffective* | 51 | 1.55 ( 1.17 - 2.04 ) | 1.53 ( 9.6 ) | 1.53 ( 1.16 ) | 0.62 ( 0.2 ) |
| headache* | 45 | 2.64 ( 1.97 - 3.55 ) | 2.61 ( 45.05 ) | 2.61 ( 1.94 ) | 1.38 ( 0.9 ) |
| myasthenia gravis* | 37 | 69.3 ( 49.88 - 96.27 ) | 68.26 ( 2391.41 ) | 66.58 ( 47.92 ) | 6.06 ( 4.13 ) |
| muscular weakness* | 35 | 7.44 ( 5.33 - 10.39 ) | 7.35 ( 191.81 ) | 7.33 ( 5.25 ) | 2.87 ( 2.15 ) |
| therapeutic response shortened* | 34 | 37.66 ( 26.78 - 52.96 ) | 37.15 ( 1180.05 ) | 36.65 ( 26.07 ) | 5.2 ( 3.69 ) |
| malaise* | 32 | 1.88 ( 1.33 - 2.67 ) | 1.87 ( 13.02 ) | 1.87 ( 1.32 ) | 0.9 ( 0.36 ) |
| diarrhoea | 32 | 0.95 ( 0.67 - 1.35 ) | 0.95 ( 0.07 ) | 0.95 ( 0.67 ) | -0.07 ( -0.57 ) |
| diplopia* | 31 | 36.34 ( 25.44 - 51.92 ) | 35.9 ( 1037.97 ) | 35.43 ( 24.8 ) | 5.15 ( 3.58 ) |
| dyspnoea | 30 | 1.1 ( 0.77 - 1.58 ) | 1.1 ( 0.28 ) | 1.1 ( 0.77 ) | 0.14 ( -0.39 ) |
| back pain* | 30 | 3.14 ( 2.19 - 4.5 ) | 3.12 ( 43.22 ) | 3.11 ( 2.17 ) | 1.64 ( 1.02 ) |
| fall | 28 | 1.2 ( 0.83 - 1.74 ) | 1.2 ( 0.93 ) | 1.2 ( 0.83 ) | 0.26 ( -0.29 ) |
| covid-19* | 24 | 1.56 ( 1.05 - 2.34 ) | 1.56 ( 4.81 ) | 1.56 ( 1.04 ) | 0.64 ( 0.03 ) |
| eyelid ptosis* | 22 | 72.05 ( 47.09 - 110.25 ) | 71.41 ( 1487.65 ) | 69.57 ( 45.47 ) | 6.12 ( 3.51 ) |
| haemoglobin decreased* | 22 | 4.03 ( 2.65 - 6.14 ) | 4.01 ( 49.69 ) | 4 ( 2.63 ) | 2 ( 1.22 ) |
| pneumonia | 21 | 1.11 ( 0.72 - 1.7 ) | 1.11 ( 0.22 ) | 1.11 ( 0.72 ) | 0.15 ( -0.48 ) |
| haemolysis* | 21 | 112.62 ( 72.65 - 174.58 ) | 111.66 ( 2210.38 ) | 107.2 ( 69.15 ) | 6.74 ( 3.57 ) |
| dizziness | 21 | 1.04 ( 0.67 - 1.59 ) | 1.04 ( 0.03 ) | 1.04 ( 0.67 ) | 0.05 ( -0.57 ) |
| gait disturbance* | 20 | 2.3 ( 1.48 - 3.57 ) | 2.29 ( 14.57 ) | 2.29 ( 1.47 ) | 1.19 ( 0.48 ) |
| dysphagia* | 20 | 4.86 ( 3.13 - 7.55 ) | 4.83 ( 60.76 ) | 4.82 ( 3.11 ) | 2.27 ( 1.4 ) |
| anaemia* | 19 | 1.66 ( 1.06 - 2.6 ) | 1.65 ( 4.92 ) | 1.65 ( 1.05 ) | 0.72 ( 0.03 ) |
| pain | 19 | 1.03 ( 0.66 - 1.62 ) | 1.03 ( 0.02 ) | 1.03 ( 0.66 ) | 0.04 ( -0.61 ) |
| feeling abnormal* | 19 | 2.53 ( 1.61 - 3.98 ) | 2.52 ( 17.49 ) | 2.52 ( 1.6 ) | 1.33 ( 0.58 ) |
| pain in extremity* | 19 | 1.65 ( 1.05 - 2.6 ) | 1.65 ( 4.87 ) | 1.65 ( 1.05 ) | 0.72 ( 0.03 ) |
| arthralgia | 17 | 1.11 ( 0.69 - 1.78 ) | 1.11 ( 0.17 ) | 1.11 ( 0.69 ) | 0.14 ( -0.55 ) |
| weight decreased | 17 | 1.3 ( 0.81 - 2.1 ) | 1.3 ( 1.2 ) | 1.3 ( 0.81 ) | 0.38 ( -0.33 ) |
| myasthenia gravis crisis* | 17 | 252.84 ( 153.52 - 416.4 ) | 251.09 ( 3869.22 ) | 229.5 ( 139.35 ) | 7.84 ( 3.35 ) |
| illness* | 16 | 2.52 ( 1.54 - 4.12 ) | 2.51 ( 14.54 ) | 2.51 ( 1.53 ) | 1.33 ( 0.5 ) |
| somnolence* | 15 | 2.19 ( 1.32 - 3.64 ) | 2.18 ( 9.64 ) | 2.18 ( 1.31 ) | 1.13 ( 0.3 ) |
| infection* | 15 | 2.7 ( 1.62 - 4.48 ) | 2.69 ( 15.92 ) | 2.69 ( 1.62 ) | 1.43 ( 0.56 ) |
| nasopharyngitis* | 15 | 2.21 ( 1.33 - 3.67 ) | 2.2 ( 9.83 ) | 2.2 ( 1.32 ) | 1.14 ( 0.31 ) |
| speech disorder* | 15 | 6.91 ( 4.16 - 11.49 ) | 6.87 ( 75.17 ) | 6.86 ( 4.13 ) | 2.78 ( 1.6 ) |
| urinary tract infection | 15 | 1.45 ( 0.87 - 2.41 ) | 1.45 ( 2.1 ) | 1.45 ( 0.87 ) | 0.54 ( -0.23 ) |
| balance disorder* | 14 | 3.24 ( 1.92 - 5.48 ) | 3.23 ( 21.54 ) | 3.23 ( 1.91 ) | 1.69 ( 0.74 ) |
| cough | 13 | 0.95 ( 0.55 - 1.64 ) | 0.95 ( 0.04 ) | 0.95 ( 0.55 ) | -0.08 ( -0.84 ) |
| condition aggravated | 13 | 1.09 ( 0.63 - 1.87 ) | 1.08 ( 0.09 ) | 1.08 ( 0.63 ) | 0.12 ( -0.67 ) |
| vomiting | 12 | 0.75 ( 0.42 - 1.31 ) | 0.75 ( 1.04 ) | 0.75 ( 0.42 ) | -0.42 ( -1.2 ) |
| pneumonia aspiration* | 12 | 7.18 ( 4.07 - 12.67 ) | 7.15 ( 63.35 ) | 7.13 ( 4.04 ) | 2.83 ( 1.47 ) |
| dyspnoea exertional* | 12 | 4.4 ( 2.49 - 7.76 ) | 4.38 ( 31.33 ) | 4.38 ( 2.48 ) | 2.13 ( 0.99 ) |
| sepsis* | 11 | 1.94 ( 1.07 - 3.52 ) | 1.94 ( 5.01 ) | 1.94 ( 1.07 ) | 0.96 ( 0.01 ) |
| insomnia | 11 | 1.37 ( 0.76 - 2.48 ) | 1.37 ( 1.09 ) | 1.37 ( 0.76 ) | 0.45 ( -0.43 ) |
| dysphonia* | 11 | 3.48 ( 1.92 - 6.3 ) | 3.47 ( 19.33 ) | 3.47 ( 1.92 ) | 1.79 ( 0.69 ) |
| platelet count decreased* | 11 | 1.82 ( 1.01 - 3.3 ) | 1.82 ( 4.07 ) | 1.82 ( 1.01 ) | 0.86 ( -0.07 ) |
| general physical health deterioration | 11 | 1.55 ( 0.86 - 2.81 ) | 1.55 ( 2.16 ) | 1.55 ( 0.86 ) | 0.63 ( -0.27 ) |
| pyrexia | 10 | 0.76 ( 0.41 - 1.42 ) | 0.77 ( 0.72 ) | 0.77 ( 0.41 ) | -0.39 ( -1.23 ) |
| chills* | 10 | 2.11 ( 1.14 - 3.93 ) | 2.11 ( 5.84 ) | 2.11 ( 1.13 ) | 1.08 ( 0.06 ) |
| off label use | 10 | 0.23 ( 0.12 - 0.42 ) | 0.23 ( 26.55 ) | 0.23 ( 0.12 ) | -2.13 ( -2.9 ) |
| symptom recurrence* | 10 | 34.31 ( 18.36 - 64.1 ) | 34.17 ( 317.96 ) | 33.75 ( 18.06 ) | 5.08 ( 2.21 ) |

Abbreviation: Asterisks (*) indicate statistically significant signals in algorithm; ROR, reporting odds ratio; PRR, proportional reporting ratio; EBGM, empirical Bayesian geometric mean; EBGM05, the lower limit of the 95% CI of EBGM; IC, information component; IC025, the lower limit of the 95% CI of the IC; CI, confidence interval; PT, preferred term.

Supplementary Table 8:

Top 50 most frequent adverse events for Ravulizumab at the PT level reported by medical personnels from FAERS data

| PT | Case numbers | ROR(95%CI) | PRR(χ^2^) | EBGM(EBGM05) | IC(IC025) |
| --- | --- | --- | --- | --- | --- |
| off label use* | 302 | 2.35 ( 2.09 - 2.63 ) | 2.27 ( 220.59 ) | 2.27 ( 2.02 ) | 1.18 ( 1.01 ) |
| myasthenia gravis* | 191 | 181.23 ( 156.09 - 210.42 ) | 175.22 ( 30798.5 ) | 163.14 ( 140.51 ) | 7.35 ( 6.25 ) |
| drug ineffective* | 138 | 1.22 ( 1.03 - 1.44 ) | 1.21 ( 5.32 ) | 1.21 ( 1.03 ) | 0.28 ( 0.03 ) |
| fatigue* | 121 | 2.08 ( 1.74 - 2.49 ) | 2.06 ( 66.31 ) | 2.06 ( 1.72 ) | 1.04 ( 0.76 ) |
| symptom recurrence* | 109 | 147.59 ( 121.42 - 179.4 ) | 144.8 ( 14664.81 ) | 136.46 ( 112.26 ) | 7.09 ( 5.65 ) |
| death* | 99 | 1.37 ( 1.12 - 1.67 ) | 1.36 ( 9.63 ) | 1.36 ( 1.12 ) | 0.45 ( 0.15 ) |
| haemolysis* | 97 | 120.19 ( 97.85 - 147.63 ) | 118.17 ( 10732.06 ) | 112.57 ( 91.65 ) | 6.81 ( 5.42 ) |
| haemoglobin decreased* | 94 | 10.72 ( 8.74 - 13.15 ) | 10.56 ( 810.94 ) | 10.51 ( 8.57 ) | 3.39 ( 2.96 ) |
| therapeutic response shortened* | 86 | 16.84 ( 13.6 - 20.85 ) | 16.6 ( 1252.91 ) | 16.49 ( 13.32 ) | 4.04 ( 3.49 ) |
| breakthrough haemolysis* | 82 | 1493.16 ( 1131.64 - 1970.17 ) | 1471.81 ( 74126.56 ) | 905.58 ( 686.33 ) | 9.82 ( 5.87 ) |
| headache* | 71 | 1.66 ( 1.31 - 2.1 ) | 1.65 ( 18.46 ) | 1.65 ( 1.31 ) | 0.73 ( 0.37 ) |
| asthenia* | 68 | 2.44 ( 1.92 - 3.09 ) | 2.42 ( 56.79 ) | 2.42 ( 1.9 ) | 1.27 ( 0.89 ) |
| extravascular haemolysis* | 66 | 2660.91 ( 1871.12 - 3784.06 ) | 2630.28 ( 81876.8 ) | 1242.02 ( 873.37 ) | 10.28 ( 5.55 ) |
| anaemia* | 65 | 2.87 ( 2.25 - 3.67 ) | 2.85 ( 78.41 ) | 2.85 ( 2.23 ) | 1.51 ( 1.11 ) |
| dyspnoea* | 64 | 1.32 ( 1.03 - 1.68 ) | 1.31 ( 4.81 ) | 1.31 ( 1.03 ) | 0.39 ( 0.03 ) |
| pyrexia* | 55 | 1.49 ( 1.14 - 1.94 ) | 1.48 ( 8.64 ) | 1.48 ( 1.14 ) | 0.57 ( 0.17 ) |
| condition aggravated | 53 | 1.26 ( 0.96 - 1.65 ) | 1.25 ( 2.75 ) | 1.25 ( 0.96 ) | 0.33 ( -0.07 ) |
| covid-19* | 51 | 1.68 ( 1.28 - 2.22 ) | 1.68 ( 13.94 ) | 1.67 ( 1.27 ) | 0.74 ( 0.32 ) |
| blood lactate dehydrogenase increased* | 48 | 29.09 ( 21.86 - 38.72 ) | 28.86 ( 1275.56 ) | 28.52 ( 21.43 ) | 4.83 ( 3.77 ) |
| infection* | 47 | 2.86 ( 2.14 - 3.81 ) | 2.84 ( 56.21 ) | 2.84 ( 2.13 ) | 1.51 ( 1.03 ) |
| malaise* | 46 | 1.56 ( 1.17 - 2.09 ) | 1.56 ( 9.27 ) | 1.56 ( 1.17 ) | 0.64 ( 0.2 ) |
| back pain* | 45 | 2.74 ( 2.05 - 3.68 ) | 2.73 ( 49.41 ) | 2.73 ( 2.03 ) | 1.45 ( 0.97 ) |
| muscular weakness* | 40 | 4.98 ( 3.64 - 6.79 ) | 4.95 ( 125.92 ) | 4.94 ( 3.62 ) | 2.3 ( 1.72 ) |
| myasthenia gravis crisis* | 38 | 198.99 ( 142.76 - 277.36 ) | 197.68 ( 6859.48 ) | 182.42 ( 130.88 ) | 7.51 ( 4.53 ) |
| nausea* | 37 | 0.6 ( 0.43 - 0.83 ) | 0.6 ( 9.76 ) | 0.6 ( 0.44 ) | -0.73 ( -1.18 ) |
| dysphagia* | 35 | 4.29 ( 3.07 - 5.98 ) | 4.27 ( 87.55 ) | 4.26 ( 3.06 ) | 2.09 ( 1.48 ) |
| pneumonia | 34 | 1.01 ( 0.72 - 1.41 ) | 1.01 ( 0 ) | 1.01 ( 0.72 ) | 0.01 ( -0.48 ) |
| platelet count decreased* | 34 | 2.97 ( 2.12 - 4.16 ) | 2.96 ( 44.17 ) | 2.96 ( 2.11 ) | 1.56 ( 1 ) |
| infusion related reaction* | 34 | 2.86 ( 2.04 - 4 ) | 2.85 ( 40.76 ) | 2.84 ( 2.03 ) | 1.51 ( 0.94 ) |
| diplopia* | 33 | 16.88 ( 11.98 - 23.8 ) | 16.79 ( 486.77 ) | 16.68 ( 11.83 ) | 4.06 ( 3.01 ) |
| eyelid ptosis* | 31 | 36.47 ( 25.56 - 52.05 ) | 36.28 ( 1047.59 ) | 35.75 ( 25.05 ) | 5.16 ( 3.58 ) |
| sepsis* | 30 | 2.33 ( 1.63 - 3.34 ) | 2.33 ( 22.7 ) | 2.32 ( 1.62 ) | 1.22 ( 0.64 ) |
| exposure during pregnancy* | 30 | 3.37 ( 2.36 - 4.83 ) | 3.36 ( 49.77 ) | 3.36 ( 2.34 ) | 1.75 ( 1.12 ) |
| urinary tract infection* | 28 | 1.91 ( 1.32 - 2.77 ) | 1.91 ( 12.12 ) | 1.91 ( 1.32 ) | 0.93 ( 0.35 ) |
| feeling abnormal* | 27 | 2.64 ( 1.81 - 3.85 ) | 2.63 ( 27.33 ) | 2.63 ( 1.8 ) | 1.39 ( 0.77 ) |
| rash | 26 | 0.54 ( 0.37 - 0.79 ) | 0.54 ( 10.1 ) | 0.54 ( 0.37 ) | -0.88 ( -1.41 ) |
| diarrhoea | 26 | 0.44 ( 0.3 - 0.65 ) | 0.44 ( 18.43 ) | 0.44 ( 0.3 ) | -1.18 ( -1.7 ) |
| blood pressure increased* | 26 | 2.04 ( 1.39 - 3 ) | 2.03 ( 13.69 ) | 2.03 ( 1.38 ) | 1.02 ( 0.41 ) |
| fall | 25 | 1.01 ( 0.68 - 1.49 ) | 1.01 ( 0 ) | 1.01 ( 0.68 ) | 0.01 ( -0.56 ) |
| thrombocytopenia | 25 | 1.43 ( 0.96 - 2.11 ) | 1.42 ( 3.17 ) | 1.42 ( 0.96 ) | 0.51 ( -0.08 ) |
| neuromyelitis optica spectrum disorder* | 25 | 92.68 ( 62.1 - 138.33 ) | 92.28 ( 2172.14 ) | 88.83 ( 59.52 ) | 6.47 ( 3.76 ) |
| arthralgia | 22 | 0.56 ( 0.37 - 0.85 ) | 0.56 ( 7.71 ) | 0.56 ( 0.37 ) | -0.84 ( -1.41 ) |
| chest pain* | 22 | 1.66 ( 1.1 - 2.53 ) | 1.66 ( 5.82 ) | 1.66 ( 1.09 ) | 0.73 ( 0.09 ) |
| abdominal pain | 22 | 1.01 ( 0.66 - 1.54 ) | 1.01 ( 0 ) | 1.01 ( 0.66 ) | 0.01 ( -0.59 ) |
| nasopharyngitis | 22 | 1.46 ( 0.96 - 2.23 ) | 1.46 ( 3.23 ) | 1.46 ( 0.96 ) | 0.55 ( -0.08 ) |
| pain | 21 | 0.53 ( 0.34 - 0.81 ) | 0.53 ( 8.84 ) | 0.53 ( 0.34 ) | -0.92 ( -1.5 ) |
| pruritus | 21 | 0.51 ( 0.34 - 0.79 ) | 0.52 ( 9.58 ) | 0.52 ( 0.34 ) | -0.95 ( -1.54 ) |
| cough | 21 | 0.9 ( 0.59 - 1.38 ) | 0.9 ( 0.24 ) | 0.9 ( 0.59 ) | -0.15 ( -0.76 ) |
| suspected covid-19* | 21 | 37.2 ( 24.16 - 57.3 ) | 37.07 ( 725.67 ) | 36.51 ( 23.71 ) | 5.19 ( 3.18 ) |
| reticulocyte count increased* | 21 | 270.81 ( 172.28 - 425.7 ) | 269.82 ( 5045.44 ) | 242.15 ( 154.04 ) | 7.92 ( 3.69 ) |

Abbreviation: Asterisks (*) indicate statistically significant signals in algorithm; ROR, reporting odds ratio; PRR, proportional reporting ratio; EBGM, empirical Bayesian geometric mean; EBGM05, the lower limit of the 95% CI of EBGM; IC, information component; IC025, the lower limit of the 95% CI of the IC; CI, confidence interval; PT, preferred term.

Supplementary Table 9:

Top 50 most frequent adverse events for Ravulizumab at the PT level reported by non-medical personnels from FAERS data

| PT | Case numbers | ROR(95%CI) | PRR(χ^2^) | EBGM(EBGM05) | IC(IC025) |
| --- | --- | --- | --- | --- | --- |
| fatigue* | 938 | 5.08 ( 4.75 - 5.43 ) | 4.79 ( 2842.64 ) | 4.77 ( 4.47 ) | 2.25 ( 2.15 ) |
| asthenia* | 449 | 5.84 ( 5.31 - 6.41 ) | 5.67 ( 1728.84 ) | 5.65 ( 5.14 ) | 2.5 ( 2.34 ) |
| headache* | 424 | 3.13 ( 2.84 - 3.44 ) | 3.06 ( 591.8 ) | 3.05 ( 2.77 ) | 1.61 ( 1.46 ) |
| drug ineffective | 273 | 0.79 ( 0.7 - 0.89 ) | 0.79 ( 15.26 ) | 0.79 ( 0.7 ) | -0.34 ( -0.51 ) |
| muscular weakness* | 264 | 11.98 ( 10.6 - 13.54 ) | 11.76 ( 2575.43 ) | 11.64 ( 10.3 ) | 3.54 ( 3.3 ) |
| malaise* | 203 | 2.13 ( 1.85 - 2.45 ) | 2.11 ( 119.42 ) | 2.11 ( 1.84 ) | 1.08 ( 0.87 ) |
| dyspnoea* | 197 | 1.85 ( 1.61 - 2.13 ) | 1.83 ( 75.34 ) | 1.83 ( 1.59 ) | 0.87 ( 0.66 ) |
| diplopia* | 192 | 40.09 ( 34.67 - 46.34 ) | 39.51 ( 6952.65 ) | 38.14 ( 32.99 ) | 5.25 ( 4.79 ) |
| back pain* | 190 | 3.77 ( 3.27 - 4.36 ) | 3.73 ( 380.6 ) | 3.73 ( 3.23 ) | 1.9 ( 1.67 ) |
| pain | 188 | 0.95 ( 0.82 - 1.09 ) | 0.95 ( 0.54 ) | 0.95 ( 0.82 ) | -0.08 ( -0.29 ) |
| feeling abnormal* | 187 | 2.92 ( 2.53 - 3.38 ) | 2.9 ( 232.51 ) | 2.89 ( 2.5 ) | 1.53 ( 1.3 ) |
| nausea | 169 | 1.09 ( 0.94 - 1.27 ) | 1.09 ( 1.27 ) | 1.09 ( 0.94 ) | 0.12 ( -0.1 ) |
| diarrhoea* | 169 | 1.22 ( 1.05 - 1.42 ) | 1.22 ( 6.64 ) | 1.22 ( 1.05 ) | 0.28 ( 0.06 ) |
| haemoglobin decreased* | 161 | 9.44 ( 8.08 - 11.04 ) | 9.34 ( 1190.32 ) | 9.27 ( 7.93 ) | 3.21 ( 2.91 ) |
| dizziness* | 151 | 1.42 ( 1.21 - 1.67 ) | 1.41 ( 18.39 ) | 1.41 ( 1.2 ) | 0.5 ( 0.26 ) |
| arthralgia* | 150 | 1.61 ( 1.37 - 1.89 ) | 1.6 ( 34.35 ) | 1.6 ( 1.36 ) | 0.68 ( 0.44 ) |
| pain in extremity* | 140 | 2.13 ( 1.8 - 2.52 ) | 2.12 ( 83.02 ) | 2.12 ( 1.79 ) | 1.08 ( 0.83 ) |
| therapeutic response shortened* | 133 | 12.97 ( 10.92 - 15.4 ) | 12.85 ( 1437.1 ) | 12.71 ( 10.7 ) | 3.67 ( 3.29 ) |
| eyelid ptosis* | 128 | 74.22 ( 62 - 88.86 ) | 73.51 ( 8565.79 ) | 68.83 ( 57.5 ) | 6.11 ( 5.23 ) |
| gait disturbance* | 119 | 2.46 ( 2.05 - 2.95 ) | 2.45 ( 102.02 ) | 2.44 ( 2.04 ) | 1.29 ( 1.01 ) |
| nasopharyngitis* | 114 | 2.59 ( 2.15 - 3.11 ) | 2.57 ( 109.68 ) | 2.57 ( 2.14 ) | 1.36 ( 1.07 ) |
| dysphagia* | 114 | 6.86 ( 5.7 - 8.25 ) | 6.8 ( 561.57 ) | 6.77 ( 5.62 ) | 2.76 ( 2.42 ) |
| covid-19* | 111 | 1.36 ( 1.13 - 1.64 ) | 1.35 ( 10.36 ) | 1.35 ( 1.12 ) | 0.44 ( 0.16 ) |
| off label use | 102 | 0.53 ( 0.43 - 0.64 ) | 0.53 ( 42.68 ) | 0.53 ( 0.44 ) | -0.91 ( -1.19 ) |
| illness* | 96 | 1.94 ( 1.59 - 2.37 ) | 1.94 ( 43.47 ) | 1.93 ( 1.58 ) | 0.95 ( 0.64 ) |
| somnolence* | 91 | 2.18 ( 1.78 - 2.69 ) | 2.18 ( 57.93 ) | 2.17 ( 1.77 ) | 1.12 ( 0.8 ) |
| haemolysis* | 91 | 165.63 ( 132.73 - 206.68 ) | 164.49 ( 12811.61 ) | 142.64 ( 114.31 ) | 7.16 ( 5.49 ) |
| myalgia* | 85 | 2.78 ( 2.25 - 3.45 ) | 2.77 ( 96.32 ) | 2.77 ( 2.24 ) | 1.47 ( 1.13 ) |
| fall | 84 | 1.17 ( 0.94 - 1.45 ) | 1.17 ( 2.05 ) | 1.17 ( 0.94 ) | 0.22 ( -0.09 ) |
| cough | 83 | 1.2 ( 0.97 - 1.49 ) | 1.2 ( 2.77 ) | 1.2 ( 0.97 ) | 0.26 ( -0.06 ) |
| muscle spasms* | 81 | 2.09 ( 1.68 - 2.61 ) | 2.09 ( 45.89 ) | 2.08 ( 1.68 ) | 1.06 ( 0.72 ) |
| pyrexia* | 76 | 1.52 ( 1.21 - 1.9 ) | 1.51 ( 13.3 ) | 1.51 ( 1.21 ) | 0.6 ( 0.26 ) |
| weight decreased | 76 | 1.16 ( 0.93 - 1.46 ) | 1.16 ( 1.73 ) | 1.16 ( 0.93 ) | 0.22 ( -0.12 ) |
| speech disorder* | 75 | 6.27 ( 5 - 7.88 ) | 6.24 ( 328.54 ) | 6.21 ( 4.95 ) | 2.63 ( 2.21 ) |
| balance disorder* | 70 | 3.36 ( 2.65 - 4.25 ) | 3.35 ( 114.91 ) | 3.34 ( 2.64 ) | 1.74 ( 1.35 ) |
| symptom recurrence* | 68 | 26.55 ( 20.86 - 33.79 ) | 26.42 ( 1623.17 ) | 25.8 ( 20.27 ) | 4.69 ( 3.89 ) |
| stress* | 66 | 3.27 ( 2.57 - 4.17 ) | 3.26 ( 103.12 ) | 3.25 ( 2.55 ) | 1.7 ( 1.3 ) |
| visual impairment* | 66 | 1.69 ( 1.33 - 2.15 ) | 1.68 ( 18.4 ) | 1.68 ( 1.32 ) | 0.75 ( 0.38 ) |
| neck pain* | 65 | 5.16 ( 4.04 - 6.59 ) | 5.14 ( 216.11 ) | 5.12 ( 4.01 ) | 2.36 ( 1.91 ) |
| insomnia | 64 | 1.04 ( 0.81 - 1.33 ) | 1.04 ( 0.1 ) | 1.04 ( 0.81 ) | 0.06 ( -0.3 ) |
| abdominal discomfort* | 64 | 1.49 ( 1.17 - 1.91 ) | 1.49 ( 10.41 ) | 1.49 ( 1.17 ) | 0.58 ( 0.21 ) |
| urinary tract infection* | 61 | 1.69 ( 1.31 - 2.17 ) | 1.69 ( 17.02 ) | 1.68 ( 1.31 ) | 0.75 ( 0.37 ) |
| general physical health deterioration* | 61 | 3.65 ( 2.83 - 4.69 ) | 3.63 ( 116.18 ) | 3.62 ( 2.82 ) | 1.86 ( 1.43 ) |
| blood pressure increased* | 59 | 1.73 ( 1.34 - 2.23 ) | 1.72 ( 17.92 ) | 1.72 ( 1.33 ) | 0.78 ( 0.39 ) |
| anxiety | 58 | 0.74 ( 0.57 - 0.95 ) | 0.74 ( 5.4 ) | 0.74 ( 0.57 ) | -0.44 ( -0.81 ) |
| weight increased | 58 | 1.21 ( 0.94 - 1.57 ) | 1.21 ( 2.17 ) | 1.21 ( 0.94 ) | 0.28 ( -0.1 ) |
| poor venous access* | 58 | 32.68 ( 25.15 - 42.46 ) | 32.54 ( 1720.73 ) | 31.6 ( 24.32 ) | 4.98 ( 4 ) |
| musculoskeletal stiffness* | 56 | 2.82 ( 2.17 - 3.66 ) | 2.81 ( 65.14 ) | 2.8 ( 2.16 ) | 1.49 ( 1.06 ) |
| vision blurred* | 55 | 1.85 ( 1.42 - 2.41 ) | 1.85 ( 21.45 ) | 1.85 ( 1.42 ) | 0.89 ( 0.48 ) |
| condition aggravated | 55 | 0.94 ( 0.72 - 1.22 ) | 0.94 ( 0.24 ) | 0.94 ( 0.72 ) | -0.09 ( -0.48 ) |

Abbreviation: Asterisks (*) indicate statistically significant signals in algorithm; ROR, reporting odds ratio; PRR, proportional reporting ratio; EBGM, empirical Bayesian geometric mean; EBGM05, the lower limit of the 95% CI of EBGM; IC, information component; IC025, the lower limit of the 95% CI of the IC; CI, confidence interval; PT, preferred term.

Supplementary Table 10:

Top 50 most frequent adverse events for Ravulizumab excluding common medication co-usage at the PT level from FAERS data

| PT | Case numbers | ROR(95%CI) | PRR(χ^2^) | EBGM(EBGM05) | IC(IC025) |
| --- | --- | --- | --- | --- | --- |
| fatigue* | 1044 | 4.82 ( 4.53 - 5.13 ) | 4.6 ( 2968.24 ) | 4.59 ( 4.31 ) | 2.2 ( 2.1 ) |
| asthenia* | 512 | 5.33 ( 4.88 - 5.82 ) | 5.2 ( 1742.16 ) | 5.19 ( 4.75 ) | 2.38 ( 2.23 ) |
| headache* | 471 | 2.91 ( 2.65 - 3.19 ) | 2.86 ( 573.55 ) | 2.86 ( 2.61 ) | 1.51 ( 1.37 ) |
| drug ineffective | 345 | 0.84 ( 0.75 - 0.93 ) | 0.84 ( 10.57 ) | 0.84 ( 0.76 ) | -0.25 ( -0.41 ) |
| dyspnoea* | 256 | 1.69 ( 1.49 - 1.91 ) | 1.68 ( 70.39 ) | 1.68 ( 1.48 ) | 0.74 ( 0.56 ) |
| muscular weakness* | 256 | 9.22 ( 8.15 - 10.44 ) | 9.1 ( 1839.46 ) | 9.06 ( 8.01 ) | 3.18 ( 2.95 ) |
| back pain* | 236 | 3.92 ( 3.44 - 4.45 ) | 3.88 ( 504.45 ) | 3.87 ( 3.4 ) | 1.95 ( 1.75 ) |
| malaise* | 235 | 2.08 ( 1.83 - 2.37 ) | 2.07 ( 129.83 ) | 2.06 ( 1.81 ) | 1.05 ( 0.85 ) |
| death | 233 | 0.93 ( 0.82 - 1.06 ) | 0.93 ( 1.26 ) | 0.93 ( 0.82 ) | -0.11 ( -0.29 ) |
| haemoglobin decreased* | 224 | 8.7 ( 7.63 - 9.93 ) | 8.61 ( 1500.65 ) | 8.57 ( 7.51 ) | 3.1 ( 2.86 ) |
| pain | 218 | 1.12 ( 0.98 - 1.28 ) | 1.12 ( 2.68 ) | 1.12 ( 0.98 ) | 0.16 ( -0.04 ) |
| therapeutic response shortened* | 215 | 15.04 ( 13.14 - 17.21 ) | 14.87 ( 2758.31 ) | 14.74 ( 12.88 ) | 3.88 ( 3.59 ) |
| feeling abnormal* | 212 | 3.56 ( 3.11 - 4.07 ) | 3.53 ( 384.26 ) | 3.52 ( 3.07 ) | 1.82 ( 1.6 ) |
| diarrhoea | 190 | 1.01 ( 0.87 - 1.16 ) | 1.01 ( 0.01 ) | 1.01 ( 0.87 ) | 0.01 ( -0.2 ) |
| myasthenia gravis* | 185 | 65.46 ( 56.48 - 75.88 ) | 64.8 ( 11180.94 ) | 62.37 ( 53.81 ) | 5.96 ( 5.33 ) |
| nausea | 181 | 0.88 ( 0.76 - 1.02 ) | 0.88 ( 2.77 ) | 0.88 ( 0.76 ) | -0.18 ( -0.39 ) |
| diplopia* | 179 | 28.03 ( 24.16 - 32.51 ) | 27.76 ( 4542.04 ) | 27.31 ( 23.54 ) | 4.77 ( 4.36 ) |
| arthralgia* | 170 | 1.38 ( 1.18 - 1.6 ) | 1.37 ( 17.27 ) | 1.37 ( 1.18 ) | 0.46 ( 0.23 ) |
| symptom recurrence* | 159 | 55.78 ( 47.59 - 65.37 ) | 55.29 ( 8201.09 ) | 53.52 ( 45.67 ) | 5.74 ( 5.1 ) |
| covid-19* | 158 | 1.55 ( 1.33 - 1.82 ) | 1.55 ( 30.73 ) | 1.55 ( 1.32 ) | 0.63 ( 0.39 ) |
| dizziness* | 152 | 1.21 ( 1.03 - 1.42 ) | 1.21 ( 5.6 ) | 1.21 ( 1.03 ) | 0.28 ( 0.04 ) |
| off label use | 149 | 0.44 ( 0.38 - 0.52 ) | 0.45 ( 102.52 ) | 0.45 ( 0.38 ) | -1.15 ( -1.39 ) |
| pain in extremity* | 147 | 1.97 ( 1.67 - 2.31 ) | 1.96 ( 69.19 ) | 1.96 ( 1.66 ) | 0.97 ( 0.72 ) |
| gait disturbance* | 145 | 2.87 ( 2.44 - 3.38 ) | 2.86 ( 175.18 ) | 2.85 ( 2.42 ) | 1.51 ( 1.25 ) |
| nasopharyngitis* | 144 | 2.59 ( 2.2 - 3.05 ) | 2.57 ( 138.84 ) | 2.57 ( 2.18 ) | 1.36 ( 1.11 ) |
| eyelid ptosis* | 135 | 52.83 ( 44.48 - 62.74 ) | 52.44 ( 6601.83 ) | 50.85 ( 42.81 ) | 5.67 ( 4.97 ) |
| haemolysis* | 134 | 76.06 ( 63.93 - 90.5 ) | 75.5 ( 9418.3 ) | 72.22 ( 60.7 ) | 6.17 ( 5.31 ) |
| fall* | 130 | 1.47 ( 1.24 - 1.74 ) | 1.46 ( 19.26 ) | 1.46 ( 1.23 ) | 0.55 ( 0.29 ) |
| dysphagia* | 129 | 5.35 ( 4.5 - 6.36 ) | 5.32 ( 451.51 ) | 5.3 ( 4.46 ) | 2.41 ( 2.11 ) |
| illness* | 121 | 2.4 ( 2.01 - 2.87 ) | 2.39 ( 98.38 ) | 2.39 ( 2 ) | 1.26 ( 0.98 ) |
| pneumonia* | 115 | 1.22 ( 1.02 - 1.47 ) | 1.22 ( 4.67 ) | 1.22 ( 1.02 ) | 0.29 ( 0.02 ) |
| pyrexia* | 114 | 1.22 ( 1.02 - 1.47 ) | 1.22 ( 4.54 ) | 1.22 ( 1.01 ) | 0.29 ( 0.01 ) |
| muscle spasms* | 103 | 2.3 ( 1.89 - 2.79 ) | 2.29 ( 74.9 ) | 2.29 ( 1.88 ) | 1.19 ( 0.89 ) |
| urinary tract infection* | 102 | 2.06 ( 1.69 - 2.5 ) | 2.05 ( 54.96 ) | 2.05 ( 1.69 ) | 1.03 ( 0.73 ) |
| infection* | 101 | 2.26 ( 1.86 - 2.75 ) | 2.26 ( 70.78 ) | 2.26 ( 1.85 ) | 1.17 ( 0.87 ) |
| somnolence* | 100 | 1.91 ( 1.57 - 2.33 ) | 1.91 ( 43.12 ) | 1.9 ( 1.56 ) | 0.93 ( 0.63 ) |
| cough | 96 | 1.13 ( 0.92 - 1.38 ) | 1.13 ( 1.37 ) | 1.13 ( 0.92 ) | 0.17 ( -0.12 ) |
| blood pressure increased* | 96 | 2.19 ( 1.79 - 2.68 ) | 2.19 ( 61.83 ) | 2.18 ( 1.79 ) | 1.13 ( 0.82 ) |
| anaemia* | 94 | 1.94 ( 1.59 - 2.38 ) | 1.94 ( 42.72 ) | 1.94 ( 1.58 ) | 0.95 ( 0.64 ) |
| myalgia* | 90 | 2.2 ( 1.79 - 2.71 ) | 2.2 ( 58.7 ) | 2.19 ( 1.78 ) | 1.13 ( 0.81 ) |
| weight decreased | 85 | 1.09 ( 0.88 - 1.34 ) | 1.09 ( 0.58 ) | 1.09 ( 0.88 ) | 0.12 ( -0.19 ) |
| condition aggravated | 81 | 0.77 ( 0.62 - 0.96 ) | 0.78 ( 5.29 ) | 0.78 ( 0.62 ) | -0.37 ( -0.68 ) |
| general physical health deterioration* | 80 | 2.28 ( 1.83 - 2.84 ) | 2.28 ( 57.28 ) | 2.27 ( 1.83 ) | 1.19 ( 0.84 ) |
| vomiting | 79 | 0.66 ( 0.53 - 0.82 ) | 0.66 ( 14.28 ) | 0.66 ( 0.53 ) | -0.61 ( -0.92 ) |
| rash | 77 | 0.58 ( 0.47 - 0.73 ) | 0.59 ( 22.6 ) | 0.59 ( 0.47 ) | -0.77 ( -1.09 ) |
| balance disorder* | 77 | 3.52 ( 2.82 - 4.41 ) | 3.51 ( 138.18 ) | 3.51 ( 2.8 ) | 1.81 ( 1.44 ) |
| visual impairment* | 74 | 1.95 ( 1.55 - 2.45 ) | 1.95 ( 34.11 ) | 1.95 ( 1.55 ) | 0.96 ( 0.61 ) |
| blood lactate dehydrogenase increased* | 74 | 23.5 ( 18.67 - 29.57 ) | 23.41 ( 1565.08 ) | 23.09 ( 18.35 ) | 4.53 ( 3.82 ) |
| vision blurred* | 73 | 2.16 ( 1.72 - 2.72 ) | 2.16 ( 45.4 ) | 2.16 ( 1.71 ) | 1.11 ( 0.75 ) |
| platelet count decreased* | 73 | 2.35 ( 1.87 - 2.96 ) | 2.34 ( 56.31 ) | 2.34 ( 1.86 ) | 1.23 ( 0.87 ) |

Abbreviation: Asterisks (*) indicate statistically significant signals in algorithm; ROR, reporting odds ratio; PRR, proportional reporting ratio; EBGM, empirical Bayesian geometric mean; EBGM05, the lower limit of the 95% CI of EBGM; IC, information component; IC025, the lower limit of the 95% CI of the IC; CI, confidence interval; PT, preferred term.
